# Fecal microbiome and metabolome dynamics during immunotherapy-based total neoadjuvant therapy in rectal cancer: associations with treatment response and toxicity

**DOI:** 10.3389/fimmu.2026.1871586

**Published:** 2026-06-24

**Authors:** Weiqing Lu, Yaqi Wang, Jing Zhang, Yida Li, Lili Huang, Wang Yang, Shujuan Zhou, Menglong Zhou, Yajie Chen, Ruiyan Wu, Yan Wang, Hui Zhang, Juefeng Wan, Fan Xia, Zhen Zhang, Lijun Shen

**Affiliations:** 1 Department of Radiation Oncology, Fudan University Shanghai Cancer Center, Shanghai, China; 2 Department of Oncology, Shanghai Medical College, Fudan University, Shanghai, China; 3 Shanghai Clinical Research Center for Radiation Oncology, Shanghai, China; 4 Shanghai Key Laboratory of Radiation Oncology, Shanghai, China; 5 Department of Radiology, The Affiliated Suzhou Hospital of Nanjing Medical University, Suzhou Municipal Hospital, Gusu School, Nanjing Medical University, Suzhou, Jiangsu, China

**Keywords:** antitumor immunity, gut microbiota, immunotherapy-based total neoadjuvant therapy (iTNT), locally advanced rectal cancer, metabolomics, therapeutic response, toxicity

## Abstract

**Background:**

Immunotherapy-based total neoadjuvant therapy (iTNT) is a promising strategy for microsatellite-stable locally advanced rectal cancer (LARC), yet therapeutic response and treatment-related toxicity remain heterogeneous. Integrated fecal microbiome and metabolome profiling may provide non-invasive biomarkers and functional clues for optimizing iTNT.

**Methods:**

We conducted a longitudinal fecal multi-omics study using samples from patients with microsatellite-stable LARC enrolled in the TORCH trial (NCT04518280). A total of 102 fecal samples were collected before treatment, during treatment, and after completion of iTNT. Metagenomic sequencing and untargeted metabolomics were integrated to characterize longitudinal microbial and metabolic changes. We also examined baseline features associated with therapeutic response, and multi-omics signatures linked to hematologic and gastrointestinal toxicities. A murine tumor model treated with radiotherapy plus immunotherapy, with or without GABA supplementation, was used for functional testing of the response-associated metabolite.

**Results:**

iTNT induced longitudinal gut microbiome remodeling. This remodeling was characterized by altered community structure, increased alpha diversity, enhanced microbial network connectivity, enrichment of Firmicutes-associated taxa, and depletion of Bacteroidetes and Proteobacteria. Fecal metabolomic profiles also shifted during treatment, with prominent changes in amino acid-related pathways and significant concordance between microbial and metabolic profiles. Responders were enriched in several Firmicutes-associated genera, including *Ruminococcus*, *Anaerostipes*, and *Coprobacillus*. In contrast, non-responders showed enrichment of *Klebsiella* and response-associated metabolites including gamma-aminobutyric acid (GABA). Microbial functional and metabolomic pathway analyses showed convergent enrichment of arginine and proline metabolism, which includes an alternative GABA-related metabolic route. Functionally, GABA supplementation weakened the antitumor efficacy of radiotherapy plus immunotherapy and was accompanied by systemic T cell dysfunction. In addition, specific microbial taxa and fecal metabolic features were associated with hematologic toxicity and diarrhea severity, with baseline metabolites showing exploratory potential for toxicity stratification.

**Conclusion:**

This study provides a longitudinal fecal microbiome–metabolome resource for iTNT in LARC and identifies candidate microbial and metabolic features associated with treatment response and toxicity. GABA was functionally supported as a response-associated immunomodulatory metabolite, while candidate microbial functional signals warrant further mechanistic validation.

## 1 Introduction

Locally advanced rectal cancer (LARC) remains a major clinical challenge despite substantial advances in multimodality treatment (1). Total neoadjuvant therapy (TNT), which delivers systemic chemotherapy and radiotherapy before surgery, has improved disease control compared with conventional treatment strategies (2). Despite this advancement, the pathological complete response rate remains unsatisfactory, typically plateauing at approximately 30% (3, 4). With the growing preference for non-surgical management to preserve organ function, novel strategies are urgently needed to further improve durable CR rates.

More recently, immune checkpoint inhibitors have revolutionized the treatment of multiple malignancies (5). Combining radiotherapy with immune checkpoint inhibitors provides a strong rationale for immunotherapy-based TNT (iTNT), particularly in the context of organ preservation (6). Radiotherapy can enhance antitumor immunity by increasing antigen release, promoting antigen presentation, and reshaping the tumor immune microenvironment. These effects may potentially synergize with programed death protein1 (PD-1)/programed death ligand 1 (PD-L1) blockade (7). Short course radiotherapy (SCRT) is particularly attractive in this setting because of its hypofractionated schedule, logistical convenience, and potential immunologic advantages (8–10). Recent clinical studies, including the UNION study and the STELLAR II study, have shown encouraging response rates with iTNT regimens in LARC, especially the SCRT-based approach (11, 12). Our institution’s TORCH trial, which employed SCRT combined with CAPEOX chemotherapy and toripalimab achieved clinical response rates exceeding 50% (13). Nevertheless, the biological determinants underlying variable response and toxicity to iTNT remain incompletely understood, posing barriers to personalized regimens design.

The gut microbiome has emerged as an important modulator of antitumor immunity and treatment response to immunotherapy and radiotherapy (14, 15). These effects are partly mediated through microbiome-derived or microbiome-modified metabolites. Examples include short-chain fatty acids, bile acid derivatives, and tryptophan catabolites, which can regulate immune cell activation, epithelial barrier integrity, and therapy-related tissue injury (16–18). Thus, integrated fecal microbiome and metabolome profiling may provide a non-invasive window into host–microbial interactions that shape both therapeutic efficacy and toxicity. However, longitudinal studies that jointly profile microbial and metabolic dynamics during iTNT remained limited. Studies linking these changes to both response and treatment-related toxicities are scarce.

To address this gap, we conducted a longitudinal fecal multi-omics study using samples collected from patients with microsatellite-stable LARC enrolled in the TORCH trial (ClinicalTrials.gov identifier: NCT04518280) (13). By integrating metagenomic sequencing and untargeted metabolomics across key treatment timepoints, we characterized gut microbiome and metabolome dynamics during iTNT. We further investigated baseline multi-omics features associated with therapeutic response. We also functionally tested a response-associated metabolite in a radiotherapy plus PD-1 blockade mouse model. In addition, we explored microbial and metabolic signatures linked to hematologic and gastrointestinal toxicities. This study provides a longitudinal fecal multi-omics resource for iTNT and identifies candidate biomarkers and functional clues relevant to personalized treatment optimization in LARC.

## 2 Materials and methods

### 2.1 Study design and patient cohort

This study was conducted at Fudan University Shanghai Cancer Center (FUSCC) and included patients with microsatellite-stable locally advanced rectal cancer enrolled in the TORCH trial (ClinicalTrials.gov identifier: NCT04518280) between April 2021 and February 2023. The study was approved by the Ethics Committee of FUSCC (approval number: 2009224-7), and all participants provided written informed consent before sample collection. Eligible patients received SCRT-based iTNT consisting of short-course radiotherapy (25 Gy in 5 fractions), six cycles of CAPEOX chemotherapy, and toripalimab. CAPEOX consisted of oxaliplatin 130 mg/m² on day 1 and capecitabine 1,000 mg/m² twice daily on days 1–14 of each cycle; toripalimab immunochemotherapy was administered at 240 mg on day 1 of each cycle. Key exclusion criteria included prior major abdominal or digestive tract surgery, severe metabolic disorders, chronic inflammatory bowel disease, and use of antibiotics, probiotics, steroids, or immunosuppressive agents within 4 weeks before sampling. Overall, 102 fecal samples were collected across three treatment timepoints and subjected to metagenomic and untargeted metabolomic profiling.

### 2.2 Fecal sample collection and storage

Fecal samples were collected at three treatment timepoints: before initiation of iTNT (Pre), within one week after completion of short-course radiotherapy and two cycles of immunochemotherapy (Mid), and within 1–2 months after completion of the remaining four cycles of immunochemotherapy (Post). The cohort included two treatment-sequence arms. Arm-A, the consolidation sequence, received SCRT before the first two cycles of immunochemotherapy. Arm-B, the induction sequence, received SCRT after the first two cycles. Thus, both arms had completed the same treatment components at the Mid timepoint, although in different sequences. A total of 102 fecal samples were obtained, including 44 Pre-treatment samples, 37 Mid-treatment samples, and 21 Post-treatment samples. All samples were collected using sterile fecal collection kits and transferred for storage at −80 °C as soon as possible. Samples were stored with minimal freeze–thaw cycles before metagenomic sequencing and untargeted metabolomic profiling.

### 2.3 Clinical response and toxicity assessment

Therapeutic response was assessed using a comprehensive clinical and pathological evaluation because some patients underwent a watch-and-wait strategy instead of immediate surgery. Response assessment was based on postoperative pathological examination when available, pelvic MRI, colonoscopy with biopsy, digital rectal examination, and serum carcinoembryonic antigen levels. Professional oncologists performed response evaluation according to established clinical criteria for rectal cancer response evaluation and watch-and-wait management by professional oncologists (19–21). Patients who achieved pathological complete response, clinical complete response, or near-complete response were classified as responders, whereas the remaining patients were classified as non-responders.

Treatment-related toxicities occurring during iTNT and within 90 days after iTNT were recorded and graded according to the Common Terminology Criteria for Adverse Events (V. 5.0). Hematologic toxicity was assessed using white blood cell count, neutrophil count, platelet count, and hemoglobin level. For each patient, the highest toxicity grade among these four indices was used to define overall hematologic toxicity severity, with grades 0–1 classified as mild, grade 2 as moderate, and grades 3–4 as severe. Gastrointestinal toxicity was evaluated primarily according to diarrhea grade. Diarrhea grades 0–1 were classified as mild, grade 2 as moderate, and grades 3–4 as severe. For analyses comparing mild and non-mild diarrhea, patients with grade 2–4 diarrhea were grouped as the non-mild diarrhea group.

### 2.4 Metagenomic sequencing and profiling

Microbial genomic DNA was extracted from fecal samples using a DNA extraction kit (QIAGEN, 51604) according to the manufacturer’s instructions. DNA quality and integrity were assessed by agarose gel electrophoresis and Qubit dsDNA HS assay (Invitrogen, Q32854). Sequencing libraries were constructed using the TruSeq Nano DNA LT Library Preparation Kit (Illumina, FC-121-4001) and sequenced on the Illumina NovaSeq 6000 platform by LC Bio Technology Co., Ltd. (Hangzhou, China).

Raw reads were processed using cutadapt (v1.9), fqtrim (v0.94), and Bowtie2 (v2.2.0) for adapter trimming, quality filtering, and host-sequence removal (22, 23). High-quality reads were assembled using MEGAHIT (v1.2.9), followed by open reading frame prediction with MetaGeneMark (v3.26) and non-redundant unigene construction with CD-HIT (v4.6.1) (24–26). Unigene abundance was calculated as transcripts per million (TPM) based on Bowtie2 alignment.

Taxonomic annotation was performed by aligning translated unigenes against the NCBI non-redundant protein database using DIAMOND (v0.9.14), followed by lowest common ancestor-based assignment (27). Functional annotation was performed against the KEGG database using DIAMOND, and KEGG ortholog (KO) abundance was calculated by summing TPM values of corresponding unigenes.

### 2.5 Untargeted metabolomics profiling

Untargeted metabolomic profiling was performed on fecal samples using a Vanquish Flex UHPLC system coupled to a Q Exactive mass spectrometer (Thermo Fisher Scientific). Metabolites were detected in both positive and negative ion modes. Full-scan MS1 spectra were acquired at a resolution of 70,000, and MS/MS spectra were acquired at a resolution of 17,500 using data-dependent acquisition to fragment the top precursor ions. Quality-control samples were included every 10 analytical runs to monitor instrument stability. Raw LC-MS data were processed using XCMS, CAMERA, and metaX for peak detection, peak grouping, retention-time correction, and feature annotation (28–30). Metabolite annotations were generated by matching observed mass-to-charge ratios against KEGG and HMDB databases, and were further supported by isotopic distribution and an in-house fragment library when available.

### 2.6 Microbiome, metabolome, and multi-omics analyses

Microbial alpha diversity was calculated using Shannon and the Gini-Simpson indices. The Simpson metric used in this study was defined as 1 – D. Beta diversity was assessed using Bray–Curtis distances followed by non-metric multidimensional scaling. Longitudinal community differences were evaluated by PERMANOVA with permutations constrained by patient identity. Longitudinally altered taxa and metabolites were identified using microbiome multivariable associations with linear model (MaAsLin2) (31), with treatment timepoint as a fixed effect and patient identity as a random effect.

Batch effects in metabolomic data were corrected using the ComBat method before downstream analyses (32). For metabolomic profiling, unsupervised principal component analysis was used to visualize global metabolic variation across treatment timepoints. KEGG pathway enrichment was performed for significantly altered metabolites. ReporterScore analysis was used to evaluate pathway-level differences based on metagenomic KO profiles and metabolomic features (33). Procrustes analysis was applied to assess global concordance between species-level microbial profiles and untargeted metabolomic profiles (34).

Baseline response-associated microbial and metabolic features were selected using random forest models, with feature importance assessed by permutation testing using rfPermute. Associations between selected microbial taxa, KOs, metabolites, and toxicity indices were evaluated using Spearman correlation or Mantel test where appropriate. Mfuzz clustering was used to identify metabolite clusters associated with diarrhea severity. Exploratory multivariate linear regression models were constructed to evaluate the potential of baseline metabolites for hematologic toxicity stratification (35–38). To address potential clinical confounding, these models were reconstructed by including available clinical covariates. For each hematologic index, the corresponding baseline blood count was included as a covariate, together with treatment cycle completion and documented treatment modification. Treatment cycle completion was defined according to whether chemotherapy or immunotherapy was completed as scheduled. Documented treatment modification was represented by oxaliplatin dose reduction. Metabolite-derived scores were calculated using the adjusted coefficients of selected metabolite features and were transformed as sign(x) × log10(|x| + 1) for visualization. In addition, partial Mantel tests were used to evaluate associations between microbial features and toxicity indices while controlling for these clinical covariates.

### 2.7 Murine tumor model

Female C57BL/6J mice aged 6–8 weeks were subcutaneously inoculated with 5 × 10^5^ MC38 colon adenocarcinoma cells. Tumor dimensions were measured every two days, and tumor volume was calculated as (length × width²)/2. When tumors reached approximately 200–300 mm³, mice were randomized to receive radiotherapy plus anti-PD-1 treatment with or without GABA supplementation. For radiotherapy, tumor sites were locally irradiated with 5 Gy per day for five consecutive days, for a total dose of 25 Gy. The rest of the body was shielded with lead blocks. Anti-PD-1 antibody (BioXcell, BE0146) was administered intraperitoneally at 10 mg/kg starting one day after radiotherapy and then every other day. Mice in the GABA group received GABA (Sigma, A2129) in drinking water at 6 mg/mL. The dose of GABA was selected based on previously published studies demonstrating the biological activity and safety of oral GABA supplementation (39, 40). Fresh GABA solution was replaced every two days. Mice were euthanized 10 days after radiotherapy by CO_2_ inhalation using a gradual-fill method at a displacement rate of 50% chamber volume per minute. Death was confirmed by cessation of respiration and absence of reflexes before tissue collection. All animal experiments were approved by the Institutional Animal Care and Use Committee of Fudan University Shanghai Cancer Center and were performed in accordance with institutional guidelines for laboratory animal care and use (IACUC number: FUSCC-IACUC-2025854).

### 2.8 Flow cytometry

Murine spleens were harvested after treatment and mechanically dissociated through a 40-μm cell strainer to generate single-cell suspensions. Red blood cells were lysed using RBC lysis buffer (Absin, abs9101). For intracellular cytokine detection, splenocytes were stimulated with a cell activation cocktail (Biolegend, 423304) in the presence of protein transport inhibitors for 4–6 h at 37 °C. Cells were first stained with a fixable viability dye to exclude dead cells, followed by Fc receptor blockade using anti-mouse CD16/32 antibody. Surface staining was then performed using fluorochrome-conjugated antibodies against CD45, CD3, CD4, CD8, and PD-1. After fixation and permeabilization, intracellular and nuclear staining was performed for FoxP3, Ki-67, and IFN-γ. Details of antibodies, clones, fluorochromes, suppliers, and catalog numbers are provided in **Supplementary Table 1**. Appropriate unstained, single-stained, and fluorescence-minus-one controls were included for compensation and gating. Data were acquired on a CytoFLEX S flow cytometer (Beckman Coulter) and analyzed using FlowJo software (v10.10).

### 2.9 Statistical analysis

Statistical analyses were performed using R software. Comparisons between two independent groups were conducted using Student’s t test or Wilcoxon rank-sum test, depending on data distribution. For comparisons among multiple groups, one-way or two-way ANOVA was used where appropriate. For longitudinal analyses, linear mixed-effects models were applied with treatment timepoint as a fixed effect and patient identity as a random effect to account for repeated observations from the same individual (41). For microbiome and metabolome analyses, PERMANOVA was performed using the vegan package, with permutations constrained by patient identity in longitudinal comparisons. MaAsLin2 models were used to identify differentially abundant taxa or metabolites, with covariates and random effects specified according to the analysis design (31). Multiple testing correction was performed using the Benjamini–Hochberg method where applicable. ReporterScore values greater than 1.64 or less than −1.64 were considered indicative of directionally enriched pathways. Spearman correlation analysis was used to evaluate associations among microbial taxa, KOs, metabolites, and clinical indices. Random forest, ROC, and multiple linear regression analyses were used for exploratory feature prioritization and toxicity stratification rather than for construction of clinically validated prediction models. Unless otherwise specified, two-sided *P* values < 0.05 were considered statistically significant.

## 3 Results

### 3.1 iTNT induces longitudinal remodeling of the gut microbiome in locally advanced rectal cancer

**Figure 1:**
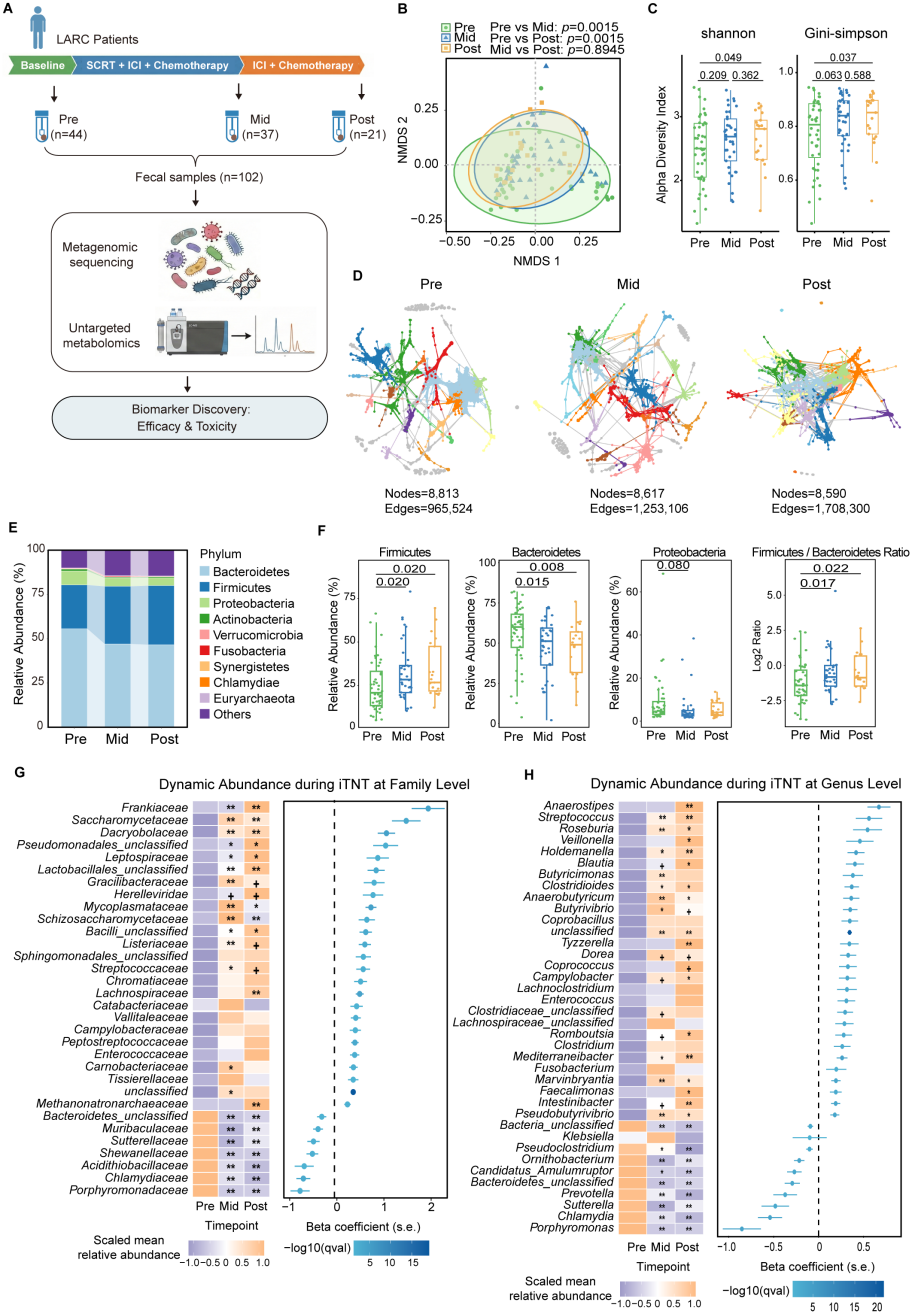
iTNT induces longitudinal remodeling of the gut microbiome in LARC patients. **(A)** Overview of the study design, treatment regimen, and fecal sample collection timepoints. Fecal samples were collected before treatment initiation (Pre), after short-course radiotherapy and two cycles of immunochemotherapy (Mid), and after completion of iTNT (Post). **(B)** NMDS plot based on Bray–Curtis distances showing longitudinal changes in gut microbial community structure across the Pre, Mid, and Post timepoints. **(C)** Alpha diversity, assessed by Shannon and Gini-Simpson indices, across the three treatment timepoints. The Gini–Simpson index was defined as 1 − D, where D = Σpi². **(D)** Microbial co-occurrence network analysis showing treatment-associated changes in microbial community organization and network complexity. **(E)** Stacked bar plots showing phylum-level microbial composition across the Pre, Mid, and Post timepoints. **(F)** Relative abundances of major phyla and the Firmicutes-to-Bacteroidetes ratio across treatment timepoints. **(G)** Longitudinally altered microbial families identified during iTNT. **(H)** Longitudinally altered microbial genera identified during iTNT. iTNT, immunotherapy-based total neoadjuvant therapy; SCRT, short-course radiotherapy; ICI, immune checkpoint inhibitors; LARC, locally advanced rectal cancer; NMDS, non-metric multidimensional scaling. + *P* < 0.1, * *P* < 0.05, ** *P* < 0.01.

To characterize the impact of immunotherapy-based total neoadjuvant therapy (iTNT) on gut microbiome, we profiled 102 longitudinal fecal samples from patients with microsatellite-stable locally advanced rectal cancer (LARC) enrolled in the TORCH trial (NCT04518280). Samples were collected at three key treatment stages: before treatment (Pre, n = 44), after short-course radiotherapy and two cycles of immunochemotherapy (Mid, n = 37), and completion of the remaining cycles of immunochemotherapy (Post, n = 21) (**Figure 1A**). The cohort included two treatment-sequence arms (Arm-A: Consolidation; Arm-B: Induction). The sample distribution from each arm across timepoints is summarized in **Supplementary Table 2**. At the Mid timepoint, both arms had completed the same treatment components, although in different sequences. Alpha diversity and community structure did not differ significantly between arms at any timepoint (**Supplementary Figure 1**). This suggested that the longitudinal microbiome patterns were unlikely to be mainly driven by treatment-sequence differences.

Longitudinal analyses, accounting for repeated measures within individuals, suggested that iTNT induced a remodeling of the gut ecosystem in LARC patients. Microbial beta diversity changed significantly during iTNT. Non-metric multidimensional scaling (NMDS) based on Bray–Curtis distances showed separation of Mid and Post samples from baseline samples (PERMANOVA constrained by patient identity, *P* < 0.05) (**Figure 1B**). Alpha diversity (Shannon and Gini-Simpson indices) exhibited a progressive increase over the treatment course (linear mixed-effects model, *P* < 0.05; **Figure 1C**). Microbial co-occurrence network analysis further suggested treatment-associated changes in community organization. Although the microbial nodes slightly decreased (Pre: 8,813 to Post: 8,590), network connectivity nearly doubled (edges Pre: 965,524 to Post: 1,708,300) (**Figure 1D**). This indicated a more interconnected microbial community after iTNT.

At the phylum level, iTNT was associated with increased Firmicutes and decreased Bacteroidetes and Proteobacteria. This led to an increased Firmicutes/Bacteroidetes ratio (linear mixed effects model, *P* < 0.05) (**Figures 1E, F**). These compositional changes were most evident between the Pre and Mid timepoints, whereas Mid and Post samples showed greater overlap. We then identified longitudinally altered taxa at lower taxonomic levels using MaAsLin2 models with patient identity as a random effect. At the family level, iTNT was associated with depletion of baseline-enriched taxa, such as Porphyromonadaceae, and enrichment of Firmicutes-associated families such as *Lachnospiraceae* and *Streptococcaceae* (adjusted *P* < 0.05) (**Figure 1G**). At the genus level, Porphyromonas and Prevotella were reduced, whereas genera commonly linked to Firmicutes-dominated gut communities, including *Anaerostipes*, *Roseburia*, and *Coprococcus*, were enriched after iTNT (adjusted *P* < 0.05) (**Figure 1H**). Species level longitudinal changes are shown in **Supplementary Figure 2**. Together, these results indicate that iTNT induces longitudinal gut microbiome remodeling in LARC patients, characterized by altered community structure, increased alpha diversity, enhanced network connectivity, and coordinated taxonomic shifts.

### 3.2 iTNT drives fecal metabolome remodeling coupled with microbial community shifts

**Figure 2:**
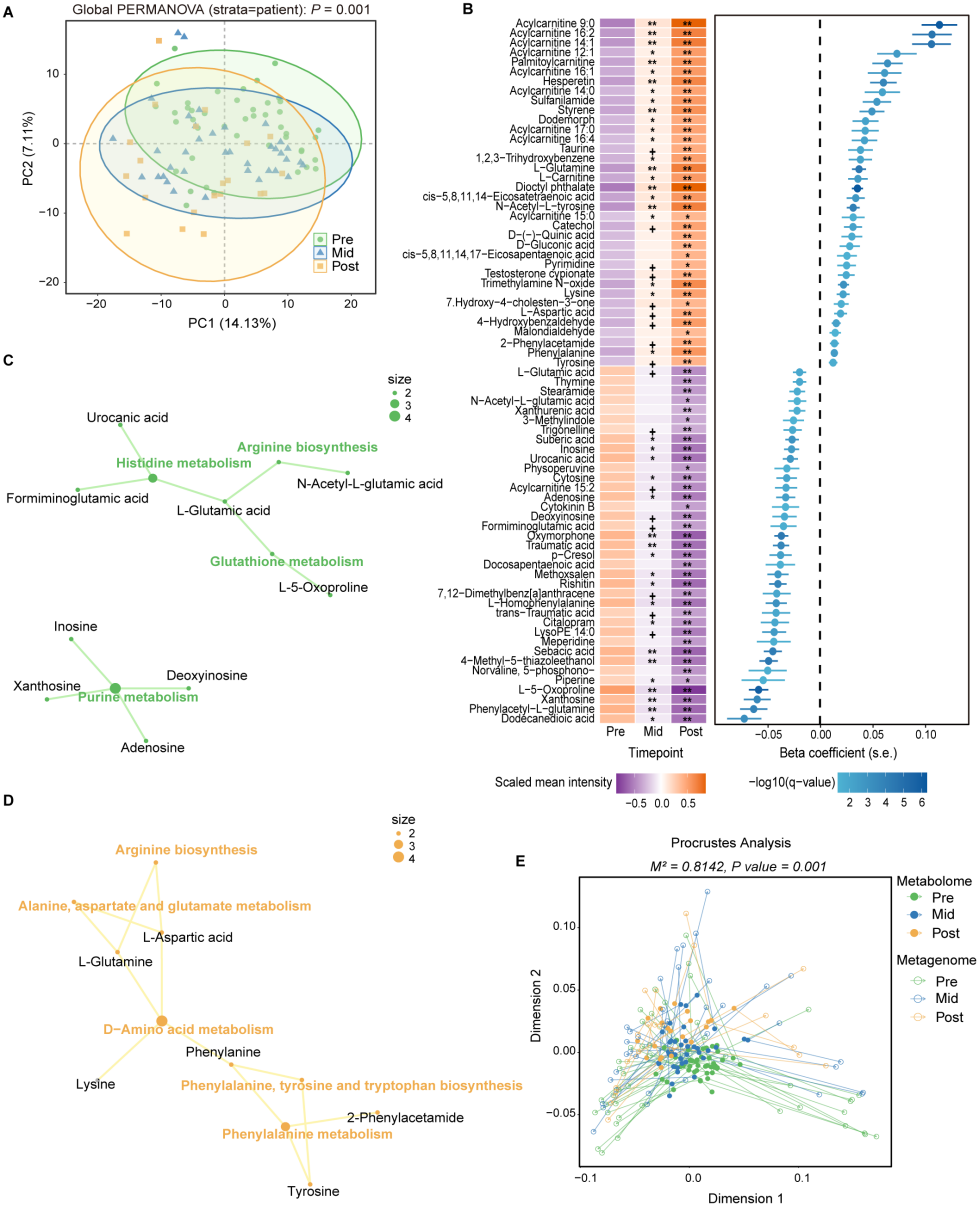
iTNT drives fecal metabolome remodeling coupled with microbial community shifts. **(A)** Unsupervised PCA of fecal metabolomic profiles across the Pre, Mid, and Post timepoints. Statistical significance was evaluated using PERMANOVA with permutations constrained by patient identity. **(B)** Heatmap of metabolites significantly altered during iTNT (adjusted *P* < 0.05), identified using MaAsLin2 with treatment timepoint as a fixed effect and patient identity as a random effect. **(C)** KEGG pathway enrichment analysis of metabolites depleted during iTNT. **(D)** KEGG pathway enrichment analysis of metabolites enriched during iTNT. **(E)** Procrustes analysis showing global concordance between species-level microbial profiles and untargeted metabolomic profiles. PCA, principal component analysis; KEGG, Kyoto Encyclopedia of Genes and Genomes. + *P* < 0.1, *P < 0.05, **P < 0.01.

We next profiled longitudinal fecal metabolites using untargeted metabolomics across the three treatment timepoints (**Figure 1A**). Unsupervised Principal Component Analysis showed a significant treatment-associated shift in the overall metabolic profile over the course of iTNT (PERMANOVA constrained by patient identity, *P* = 0.001) (**Figure 2A**). These results indicated that iTNT induced marked remodeling of fecal metabolic composition. To identify metabolites that changed during treatment, we applied MaAsLin2 models with treatment timepoint as a fixed effect and patient identity as a random effect. This analysis identified a panel of metabolites that were significantly altered over the course of iTNT (adjusted *P* < 0.05) (**Figure 2B**; **Supplementary Table 3**). Pathway enrichment analysis showed that metabolites depleted during treatment were mainly enriched in purine metabolism and histidine metabolism (**Figure 2C**). In contrast, metabolites increased during iTNT were enriched in amino acid-related pathways, including D-amino acid metabolism, alanine, aspartate and glutamate metabolism, and arginine biosynthesis (**Figure 2D**).

We further evaluated whether metabolomic remodeling was coupled with microbial community changes. Procrustes analysis based on species-level microbial profiles and untargeted metabolomic profiles showed significant concordance between the two omics layers (M² = 0.8142, *P* < 0.05) (**Figure 2E**). To explore specific targeted associations underpinning this concordance, we conducted a correlation analysis between the altered microbial genera and the key metabolites enriched in the identified pathways (**Supplementary Figure 3**). Together, these findings indicate that iTNT induces coordinated fecal metabolome remodeling alongside gut microbial shifts, with prominent changes in amino acid-related metabolic pathways.

### 3.3 Baseline fecal multi-omics features are associated with therapeutic response

We next investigated whether baseline fecal multi-omics profiles were associated with therapeutic response to iTNT. Baseline comparisons between responders (n = 29) and non-responders (n = 15) were performed to explore discriminatory biomarkers. Clinically, Responders were defined as patients who achieved a pathological or clinical complete response, whereas all other patients were classified as non-Responders. The detailed baseline information on the clinical cohort is listed in **Table 1**.

**Table 1:** Baseline characteristics of the patients with LARC enrolled in the study.

Characteristic	Overall (n = 44)	NR (n = 15)	R (n = 29)	*P* value^1^
Age, Mean (SD)	53.82 (10.81)	53.13 (11.13)	54.17 (10.82)	0.656
Sex, n (%)				0.156
Female	15.00 (34.09%)	3.00 (20.00%)	12.00 (41.38%)	
Male	29.00 (65.91%)	12.00 (80.00%)	17.00 (58.62%)	
BMI, Mean (SD)	23.46 (3.45)	23.23 (3.58)	23.57 (3.43)	0.504
Distance, Mean (SD)	3.88 (1.79)	4.13 (1.42)	3.76 (1.96)	0.345
Location, n (%)				>0.999
Middle-high	14.00 (31.82%)	5.00 (33.33%)	9.00 (31.03%)	
Low	30.00 (68.18%)	10.00 (66.67%)	20.00 (68.97%)	
Size, Mean (SD)	4.57 (1.45)	4.82 (1.23)	4.44 (1.55)	0.275
T stage, n (%)				0.226
2	4.00 (9.09%)	0.00 (0.00%)	4.00 (13.79%)	
3	37.00 (84.09%)	13.00 (86.67%)	24.00 (82.76%)	
4	3.00 (6.82%)	2.00 (13.33%)	1.00 (3.45%)	
N stage, n (%)				0.112
0	3.00 (6.82%)	0.00 (0.00%)	3.00 (10.34%)	
1	21.00 (47.73%)	5.00 (33.33%)	16.00 (55.17%)	
2	20.00 (45.45%)	10.00 (66.67%)	10.00 (34.48%)	
stage, n (%)				0.540
II	3.00 (6.82%)	0.00 (0.00%)	3.00 (10.34%)	
III	41.00 (93.18%)	15.00 (100.00%)	26.00 (89.66%)	
MRF, n (%)				0.067
0	34.00 (77.27%)	9.00 (60.00%)	25.00 (86.21%)	
1	10.00 (22.73%)	6.00 (40.00%)	4.00 (13.79%)	
EMVI, n (%)				0.235
0	35.00 (79.55%)	10.00 (66.67%)	25.00 (86.21%)	
1	9.00 (20.45%)	5.00 (33.33%)	4.00 (13.79%)	
Diarrhea, n (%)				0.818
0	1.00 (2.27%)	0.00 (0.00%)	1.00 (3.45%)	
1	20.00 (45.45%)	6.00 (40.00%)	14.00 (48.28%)	
2	19.00 (43.18%)	8.00 (53.33%)	11.00 (37.93%)	
3	4.00 (9.09%)	1.00 (6.67%)	3.00 (10.34%)	
Blood score, n (%)				0.601
0	2.00 (4.55%)	0.00 (0.00%)	2.00 (6.90%)	
1	9.00 (20.45%)	5.00 (33.33%)	4.00 (13.79%)	
2	13.00 (29.55%)	4.00 (26.67%)	9.00 (31.03%)	
3	17.00 (38.64%)	5.00 (33.33%)	12.00 (41.38%)	
4	3.00 (6.82%)	1.00 (6.67%)	2.00 (6.90%)	
WBC(x10^9^/L), Mean (SD)	3.41 (0.98)	3.79 (0.98)	3.22 (0.93)	0.107
NEU(x10^9^/L), Mean (SD)	1.82 (0.74)	2.07 (0.90)	1.69 (0.63)	0.229
PLT(x10^9^/L), Mean (SD)	78.61 (59.26)	96.60 (79.71)	69.31 (44.19)	0.322
Hb(g/L), Mean (SD)	107.55 (22.29)	111.33 (11.34)	105.59 (26.21)	0.638

^1^ Wilcoxon rank sum test; Pearson’s Chi-squared test; Fisher’s exact test.

**Figure 3:**
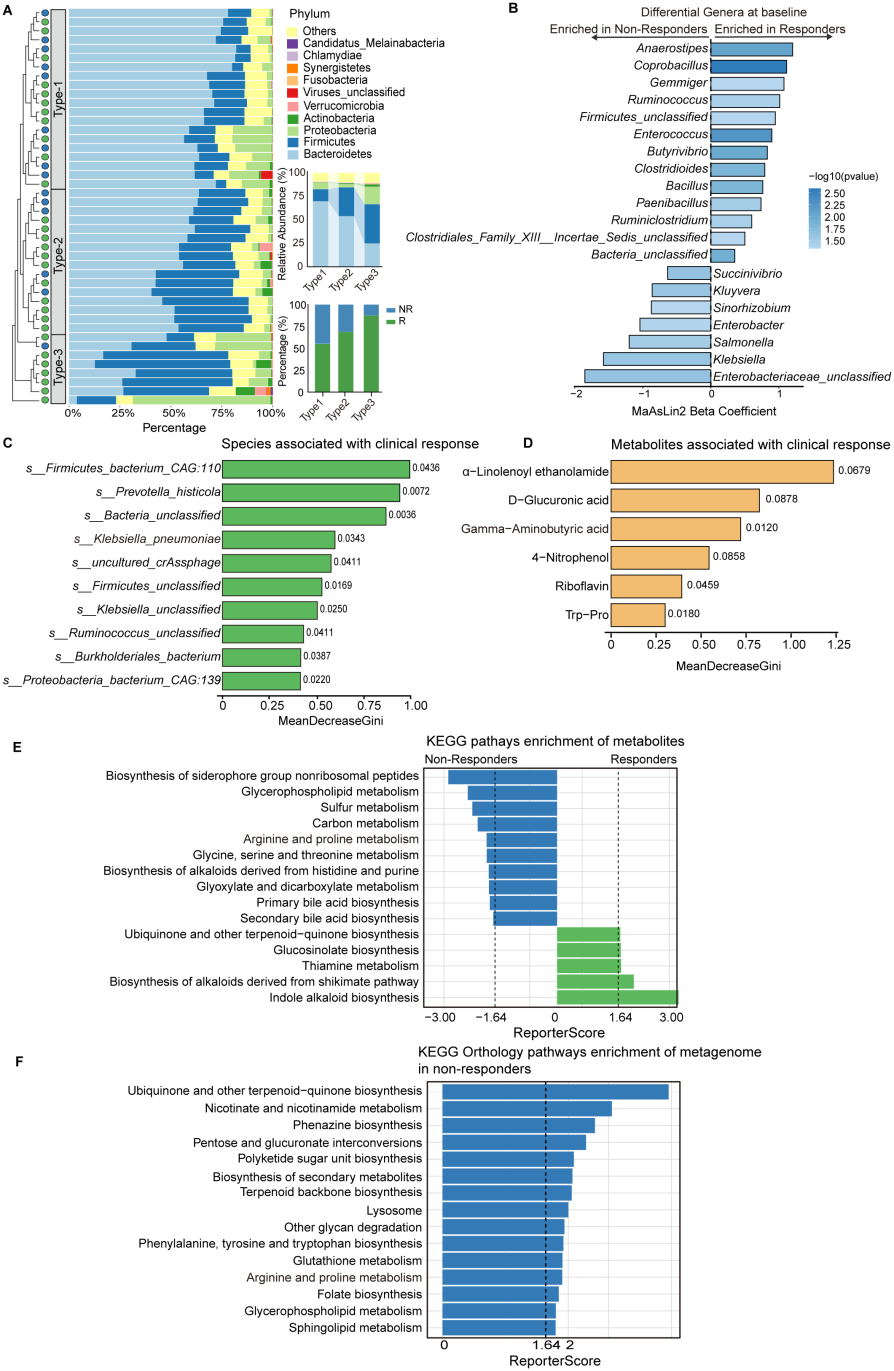
Baseline fecal multi-omics features associated with therapeutic response to iTNT. **(A)** Unsupervised clustering of baseline microbial profiles based on Bray–Curtis distances, with corresponding response status and phylum-level composition. **(B)** Differentially abundant genera between responders and non-responders identified by MaAsLin2 ((adjusted *P* < 0.05).). **(C)** Random forest-selected microbial features associated with therapeutic response. **(D)** Random forest-selected metabolic features associated with therapeutic response. **(E)** ReporterScore pathway enrichment analysis of response associated metabolomic features. **(F)** ReporterScore pathway enrichment analysis of response associated metagenomic KO profiles. ReporterScore values greater than 1.64 or less than −1.64 indicate directionally enriched pathways. KO, KEGG ortholog; RF, random forest.

Unsupervised clustering of baseline microbial profiles identified three community clusters (**Figure 3A**). Cluster 3 showed the highest proportion of responders and was characterized by higher relative abundances of Firmicutes and Actinobacteria and lower abundance of Bacteroidetes. Similar phylum-level differences were observed between responders and non-responders (**Supplementary Figure 4A**). We then used MaAsLin2 to identify specific taxa associated with response. At the genus level, responders were enriched in several Firmicutes-associated genera, including *Anaerostipes*, *Coprobacillus*, and *Ruminococcus*, whereas non-responders showed enrichment of *Klebsiella*, *Salmonella* and *Enterobacter* (adjusted *P* < 0.05) (**Figure 3B**). Species-level analysis further identified response-associated bacterial species, including enrichment of *Klebsialla pneumoniae* (*K. pneumoniar*) and *Klebsiella quasipneumoniae* in non-responders, *Ruminococcus bromii* in Responders (**Supplementary Figure 4B**). Consistently, random forest feature ranking highlighted microbial features associated with response status, with *K. pneumoniae* among the top-ranked taxa (**Figure 3C**).

We next evaluated fecal metabolites associated with response. Random forest analysis identified multiple response-associated metabolites, including gamma-aminobutyric acid (GABA), riboflavin, and Trp-Pro (**Figure 3D**). ReporterScore analysis of metabolomic data also showed pathway-level differences between responders and non-responders, with arginine and proline metabolism and glycerophospholipid metabolism enriched in non-responders (**Figure 3E**). To examine whether microbial functions showed concordant pathway differences, we performed ReporterScore analysis using metagenomic KEGG Orthology profiles. Several microbial functional pathways were enriched in non-responders, including arginine and proline metabolism and glycerophospholipid metabolism (**Figure 3F**). These overlapping KEGG pathways suggested partial concordance between baseline microbial functional potential and fecal metabolic features. Correlation analysis showed associations between several response-associated taxa and metabolites, including a positive correlation between *K. pneumoniae* and GABA (**Supplementary Figure 3C**). Exploratory receiver operating characteristic analyses showed that *K. pneumoniae* and GABA were associated with response, with AUC values of 73.8% and 68.0%, respectively. The combined model based on *K. pneumoniae* and GABA yielded an AUC value of 72.6% (**Supplementary Figure 4D**). Thus, combining the two features did not further improve response-associated discrimination in this cohort, possibly because these two features provide partially overlapping information.

Together, these results indicate that fecal microbial, and metabolic features are associated with therapeutic response to iTNT. The partial convergence of arginine/proline and glycerophospholipid related signals across metagenomic and metabolomic analyses provided a rationale for further evaluating selected response-associated metabolic features.

### 3.4 GABA supplementation attenuates radiotherapy plus anti-PD-1 efficacy and systemic T-cell immunity

**Figure 4:**
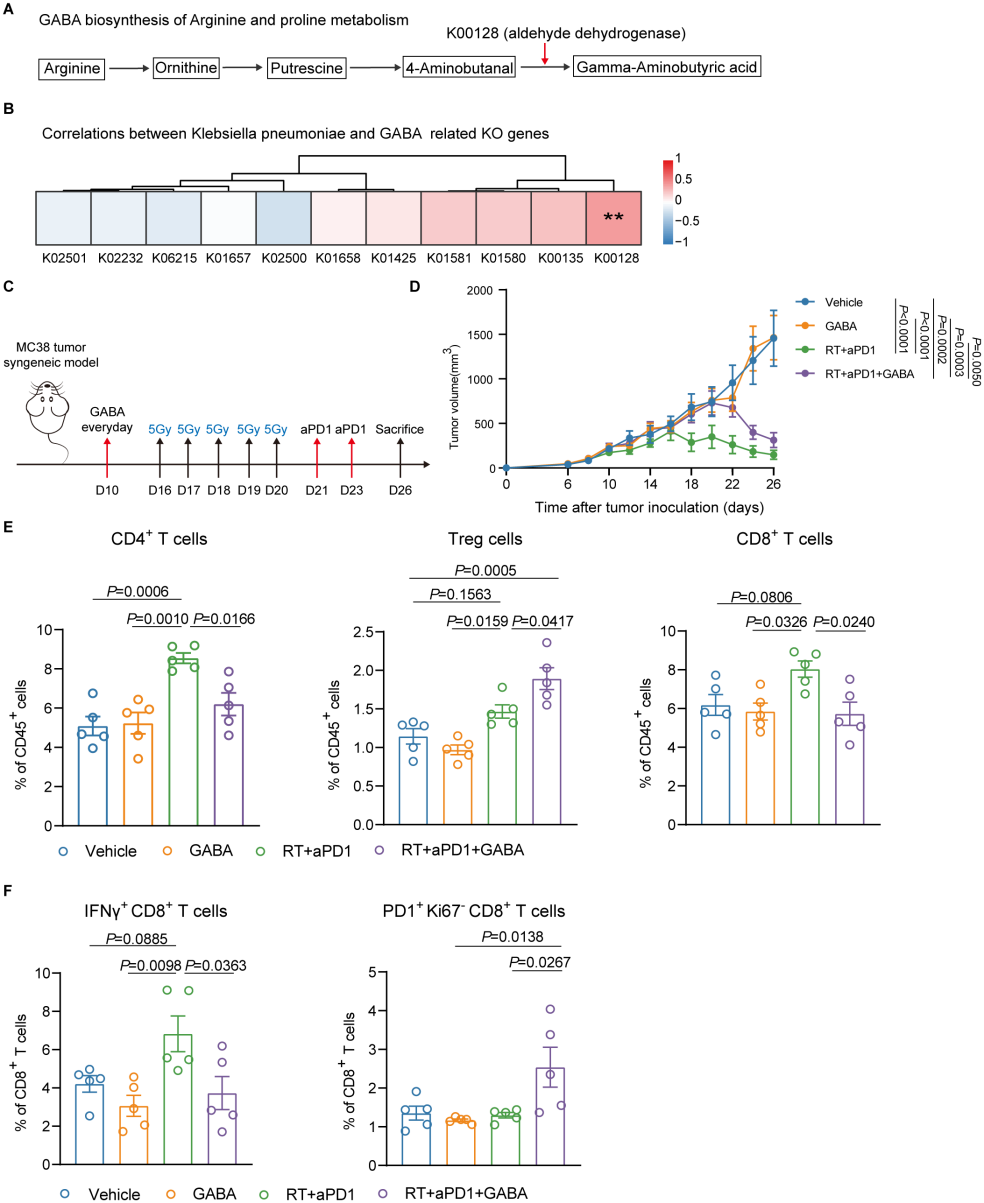
GABA-related microbial functional signals and *in vivo* functional testing of GABA. **(A)** Simplified schematic of the alternative arginine/proline-associated GABA-related metabolic route. **(B)** Correlation analysis between *K. pneumoniae* abundance and GABA-related KEGG orthologs, showing a significant positive association with K00128. **(C)** Experimental design of the MC38 tumor-bearing mouse model treated with radiotherapy plus anti-PD-1, with or without oral GABA supplementation. **(D)** Tumor growth or endpoint tumor burden in mice treated with radiotherapy plus anti-PD-1 in the presence or absence of GABA supplementation. **(E)** Flow cytometric analysis of CD4^+^ T cells, CD8^+^ T cells, and Treg cells in murine spleens after treatment. **(F)** Flow cytometric analysis of IFNγ^+^ CD8^+^ T cells and PD-1^+^Ki-67^−^ CD8+ T cells after treatment. Statistical comparisons for animal experiments were performed using one-way or two-way ANOVA as appropriate. GABA, gamma-aminobutyric acid; RT, radiotherapy; Treg, regulatory T cell.

Given that GABA was associated with therapeutic response and that arginine and proline metabolism were observed in both metagenomic and metabolomic analyses, we next examined GABA-related microbial functional features. We evaluated KEGG orthologs involved in both the classical glutamate-dependent GABA synthesis pathway and alternative arginine/proline routes. K00128, annotated as aldehyde dehydrogenase and involved in the conversion of 4-aminobutanal to GABA in the arginine/proline route (42), showed a significant positive correlation with *K. pneumoniae* abundance (**Figures 4A, B**). This association suggested a candidate microbial functional signal related to response-associated GABA in non-responders, but it did not establish the *in vivo* source of GABA.

We then established an MC38 tumor-bearing mouse model treated with focal radiotherapy and anti- PD-1, with or without oral GABA supplementation (**Figure 4C**). We found that GABA administration attenuated the tumor control effect of radiotherapy plus anti PD-1 (**Figure 4D**). We further assessed systemic T cell profiles by flow cytometry. The gating strategy and representative flow cytometry plots are shown in **Supplementary Figures 5**, **6**, respectively. Compared with mice treated with radiotherapy plus anti-PD-1 alone, mice receiving additional GABA showed reduced proportions of CD4^+^ and CD8^+^ T cells and an increased proportion of Treg cells (*P* < 0.05) (**Figure 4E**). GABA supplementation was also associated with impaired CD8^+^ T cell effector function and with increased CD8^+^ T cell exhaustion (*P* < 0.05) (**Figure 4F**). Together, these results indicate that GABA is a functionally active metabolite capable of weakening treatment-induced antitumor immunity *in vivo*. The accompanying *K. pneumoniae* association provides a candidate microbial functional context for the GABA-related phenotype. However, it should be interpreted as hypothesis-generating rather than causal evidence of microbial GABA production.

### 3.5 Specific microbial taxa are associated with iTNT-related toxicities

**Figure 5:**
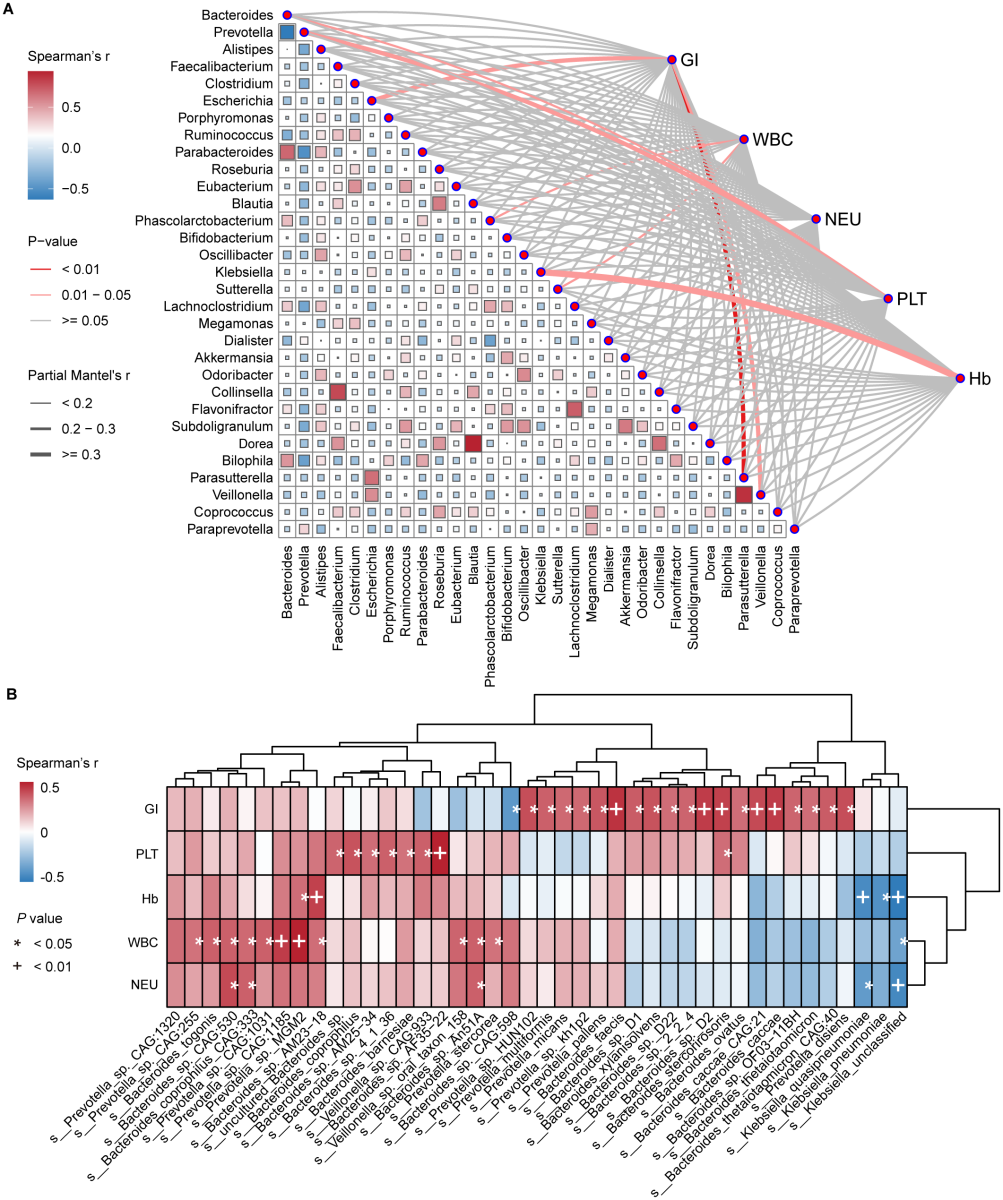
Specific microbial taxa are associated with iTNT-related toxicities. **(A)** Partial mantel test analysis showing associations between genus-level microbial features and treatment-related toxicity indices, after adjustment for available clinical covariates. **(B)** Species-level correlation heatmap showing associations between selected microbial taxa and toxicity-related clinical indices. Correlations include taxa associated with gastrointestinal toxicity and hematologic parameters, such as Klebsiella, Bacteroides, Parasutterella, and Veillonella-related features. Correlation coefficients and statistical significance are indicated in the panels. GI, gastrointestinal; Hb, hemoglobin; NEU, neutrophil.

Since iTNT may lead to adverse events while improving efficacy, we explored gut microbiome signatures associated with adverse events. We focusing on acute hematologic toxicity and gastrointestinal adverse effects, predominantly diarrhea. Overall microbial diversity did not differ significantly across toxicity grades (**Supplementary Figures 7A–D**). We then used partial mantel test analysis to assess genus-level associations with toxicity indices after adjustment for available clinical covariates (**Figure 5A**; **Supplementary Table 4**). The covariate matrix included treatment cycle completion and documented treatment modification. For hematologic toxicity, the corresponding baseline hematologic index was also included. Gastrointestinal toxicity score was associated with genera including *Escherichia*, *Parasutterella*, and *Veillonella*, with *Parasutterella* and *Veillonella* also showing positive correlations with each other. In addition, *Klebsiella* showed an association with hemoglobin-related toxicity indices. Further correlation analysis showed that *Klebsiella*-associated species, including *K. pneumoniae* and *K. quasipneumoniae*, were correlated with hematologic toxicity-related indices, particularly hemoglobin and neutrophil. Several Bacteroides species also showed associations with different toxicity indicators. Together, these findings suggest that iTNT-related toxicities are linked to specific microbial taxa rather than broad changes in microbial diversity. These associations may provide potential microbial features for toxicity risk stratification, although their mechanistic roles require further validation.

### 3.6 Baseline fecal metabolites show exploratory predictive potential for hematologic toxicity

**Figure 6:**
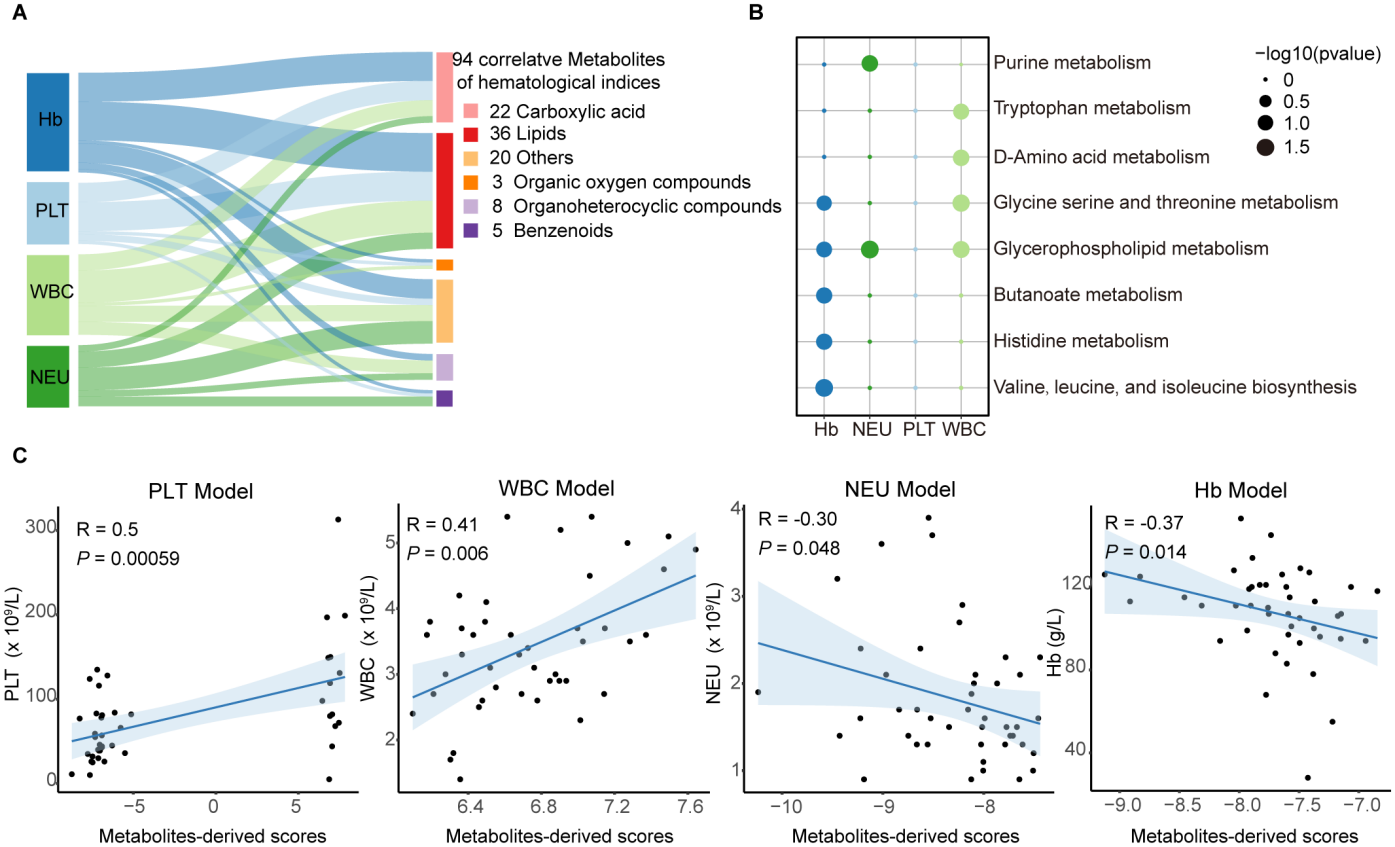
**(A)** Sankey map of fecal metabolites correlated with hematologic toxicity-related indices, including white blood cell count, neutrophil count, hemoglobin level, and platelet count. **(B)** KEGG pathway enrichment analysis of hematologic toxicity-associated metabolites. **(C)** Correlations between covariate-adjusted metabolite-derived scores and observed WBC, NEU, Hb, and PLT values during treatment. Metabolite-derived scores were calculated using the adjusted coefficients of selected metabolite features from multiple linear regression models. Scores were transformed as sign(x) × log10(|x| + 1) for visualization. WBC, white blood cell; NEU, neutrophil; Hb, hemoglobin; PLT, platelet.

We next investigated whether fecal metabolic features were associated with hematologic toxicity during iTNT. Correlation analysis identified 94 metabolites associated with at least one hematologic toxicity-related index, including white blood cells, neutrophils, hemoglobin, and platelets (|Spearman’s r| > 0.3, *P* < 0.05) (**Figure 6A**; **Supplementary Table 5**). Pathway enrichment analysis of these metabolites revealed enrichment of amino acid and lipid related metabolic pathways, including D-amino acid metabolism, tryptophan metabolism, and glycerophospholipid metabolism (**Figure 6B**). These results indicate that baseline fecal metabolic variations may reflect host–microbiome metabolic states associated with subsequent hematologic toxicity during iTNT.

To further evaluate the associations between baseline metabolites and toxicity, we constructed exploratory multiple linear regression models for hematologic indices. Metabolite subsets were selected for modeling the lowest observed levels of hematologic toxicity indices during treatment. Each model was adjusted for the corresponding baseline blood count, treatment cycle completion, and documented treatment modification. After adjustment, the β directions of the selected metabolite features were consistent with the original models. Several metabolites remained statistically significant, whereas others showed attenuated significance after adjustment (**Supplementary Figure 8**; **Supplementary Table 6**). Using the adjusted coefficients of selected metabolite features, we calculated metabolite-derived scores for WBC, NEU, Hb, and PLT. The predicted scores were significantly associated with the observed values for these hematologic indices (|Spearman’s r| > 0.3, *P* < 0.05) (**Figure 6C**). These findings suggest that fecal metabolites may have exploratory predictive potential for iTNT-related hematologic toxicity, although validation in larger independent cohorts is required.

### 3.7 Metabolic features are associated with iTNT-related diarrhea severity

**Figure 7:**
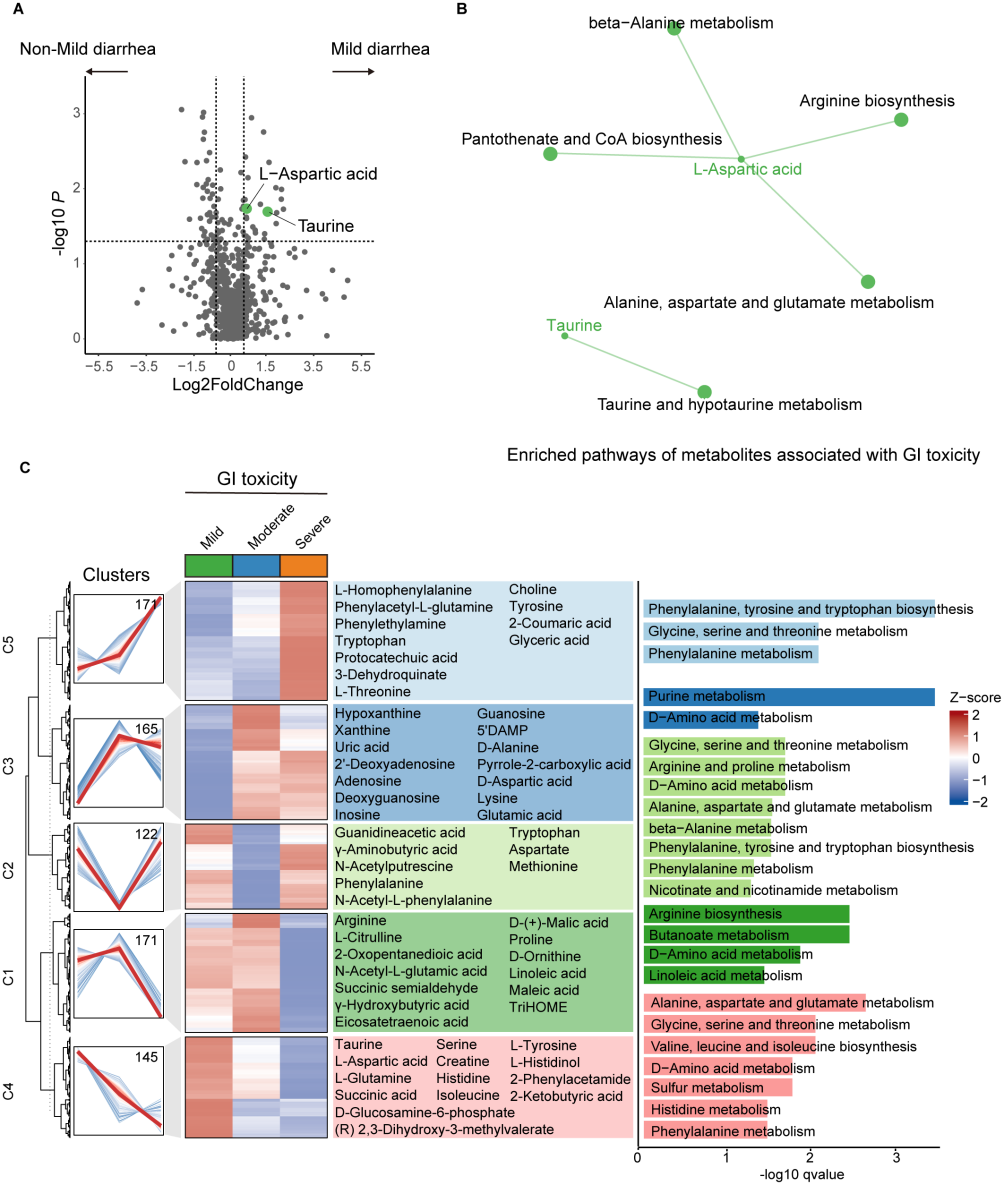
Metabolic features associated with iTNT-related diarrhea severity. **(A)** Volcano plot showing differentially abundant fecal metabolites between patients with mild diarrhea and non-mild diarrhea, with L-aspartic acid and taurine highlighted. Non-mild diarrhea was defined as grade 2–4 diarrhea. **(B)** KEGG enrichment network of diarrhea-associated metabolites and enriched metabolic pathways. **(C)** Mfuzz clustering analysis showing metabolite trajectories across increasing diarrhea severity. KEGG, Kyoto Encyclopedia of Genes and Genomes.

As diarrhea was the most common gastrointestinal toxicity in our cohort, we further explored fecal metabolic features associated with diarrhea severity. Differential metabolite analysis identified several metabolites associated with diarrhea status, among which L-aspartic acid and taurine were enriched in patients with mild diarrhea (**Figure 7A**). Pathway enrichment analysis of diarrhea-associated metabolites revealed five significantly enriched metabolic pathways (**Figure 7B**). In this enrichment network, L-aspartic acid and taurine were linked to multiple diarrhea-associated pathways, suggesting that amino acid- and taurine-related metabolic features may be involved in the metabolic differences associated with milder gastrointestinal toxicity. To capture metabolic trajectories across increasing diarrhea severity, we performed Mfuzz clustering of fecal metabolites. We identified five distinct metabolite clusters with severity-dependent patterns (**Figure 7C**). Notably, L-aspartic acid and taurine were both assigned to cluster C4, which comprised metabolites that decreased with increasing diarrhea severity. Other metabolite clusters showed progressive enrichment in patients with more severe diarrhea. These dynamic patterns further support an association between fecal metabolic state and gastrointestinal toxicity during iTNT.

## 4 Discussion

In this prospective longitudinal study, we profiled fecal microbiome–metabolome dynamics in patients with microsatellite-stable locally advanced rectal cancer receiving iTNT. We found that iTNT induced coordinated microbial and metabolic remodeling, and that baseline multi-omics features were associated with treatment response and toxicities. Functional experiments further supported GABA as a response-associated metabolite capable of weakening iTNT efficacy *in vivo*. These findings provide fecal multi-omics resource and offer candidate biomarkers for response and toxicity stratification during iTNT.

A major finding of this study is that iTNT induced broad but organized remodeling of the gut ecosystem. Unlike the microbial diversity loss reported in some long-course neoadjuvant chemoradiotherapy (nCRT) studies (43, 44), our SCRT-based iTNT cohort showed increased alpha diversity, and enhanced microbial network connectivity during treatment. Similar diversity loss was also observed in a PD-1-containing long-course nCRT cohort (45), whereas another nCRT study suggested that diversity decline during treatment was more evident in non-responders than in responders (46). This remodeling was accompanied by enrichment of Firmicutes-associated taxa, including *Lachnospiraceae* and SCFA-producing genera such as *Anaerostipes* and *Roseburia*. These findings are consistent with previous reports (43, 45, 47) and have been linked to antitumor immune modulation (48–50) (**Supplementary Table 7**). These microbial changes were accompanied by substantial metabolome remodeling, with significant concordance between microbial and metabolic profiles. This coordination suggests that iTNT imposes a distinct ecological pressure on the gut microbiome, leading to community reorganization rather than simple microbial depletion. Given the combined effects of radiotherapy, chemotherapy, and PD-1 blockade in this regimen, future studies with larger cohorts and treatment-specific sampling designs are needed to distinguish the relative contribution of each therapeutic component.

Baseline fecal multi-omics profiles also showed associations with therapeutic response. Responders were enriched in several Firmicutes-associated commensals, including *Ruminococcus*, *Anaerostipes*, and *Coprobacillus*. This observation is consistent with studies linking certain beneficial commensals, such as *Ruminococcus*, to enhanced antitumor immunity and favorable responses to immune checkpoint blockade across tumor types (15, 48). In the context of rectal cancer neoadjuvant therapy, previous studies have also linked response to these commensal taxa and fatty-acid-related microbial functions, supporting a shared theme of metabolically active beneficial microbes in treatment sensitivity (**Supplementary Table 7**) (43–46, 51). In contrast, non-responders showed enrichment of *Klebsiella* and metabolic features including GABA. At the pathway level, arginine/proline metabolism and glycerophospholipid metabolism emerged from both microbial functional and metabolomic analyses, suggesting partial concordance between microbial potential and fecal metabolic state. These findings support the concept that gut ecological and metabolic features may influence, or at least reflect, host responsiveness to immunotherapy-containing regimens (16, 52). Previous studies have reported other resistance-associated signatures, including Bacteroides, *Prevotella*, *Fusobacterium*, and metabolic programs such as nucleotide biosynthesis, suggesting that resistance-associated microbial features may differ across treatment contexts (43–45). However, direct pooled analysis with previous cohorts was not performed because of differences in sequencing platforms, treatment regimens, sampling schedules, and available metabolomic data. Independent validation cohorts will be required before these features can be applied for patient stratification in real world.

Among the metabolic features, GABA was selected for functional testing because it was significantly associated with response and correlated with *K. pneumoniae*-related microbial signatures. The positive association between *K. pneumoniae* and K00128, an aldehyde dehydrogenase involved in the alternative arginine/proline associated route to GABA, is biologically plausible given recent evidence that enteric pathobionts can use arginine, ornithine, and putrescine related pathways to generate GABA (42, 53–56). Importantly, our *in vivo* experiment supported the functional consequence of GABA. GABA supplementation weakened the antitumor efficacy of radiotherapy plus PD-1 blockade. This effect was associated with T cell dysfunction, including increased Tregs, reduced IFNγ-producing CD8^+^ T cells, and increased exhausted CD8^+^ T cells. These findings are consistent with previous evidence that GABA can promote tumor progression and immunosuppression, including suppression of CD8^+^ T cell infiltration (57). Previous studies have showed that GABA signaling can suppress effector T cell response, promote regulatory immune phenotypes, and attenuate antitumor activity. Mechanistically, these effects may be mediated, at least in part, through GABA receptor signaling on immune cells (58–60). Thus, elevated GABA levels may blunt the synergistic anti-tumor immunity induced by radiotherapy and PD-1 blockade. Nevertheless, these data should not be interpreted as proof that *K. pneumoniae* is the dominant *in vivo* source of GABA. The *in vivo* source of fecal GABA in patients remains unresolved and may include microbial, host, and dietary contributions. Therefore, GABA should be viewed as a response-associated metabolite rather than a microbiota-derived metabolite in this study. Instead, *K. pneumoniae* and K00128 should be viewed as candidate upstream microbial functional signals that warrant future validation using bacterial colonization, strain-level functional assays, or isotope-tracing approaches.

Beyond therapeutic response, our study also suggests that fecal multi-omics features may be informative for iTNT-related toxicity. Although global microbial diversity did not differ by toxicity severity, specific taxa were associated with hematologic and gastrointestinal toxicity indices, indicating that taxa-specific signals may be more informative. Fecal metabolites showed stronger associations with toxicity phenotypes, particularly for hematologic indices and diarrhea severity. The exploratory multiple linear regression models further suggested that baseline metabolic profiles may help stratify hematologic toxicity risk, although these models require independent validation. For gastrointestinal toxicity, L-aspartic acid and taurine were enriched in patients with mild diarrhea and decreased with increasing diarrhea severity, suggesting that they may mark a metabolic state associated with lower gastrointestinal toxicity burden. This observation is consistent with previous studies linking amino acid metabolism, including L-aspartate related pathways, to intestinal barrier and inflammatory regulation (61–63). These findings support the potential value of fecal metabolomics for toxicity risk stratification during iTNT, while functional validation is needed to determine whether these metabolites directly modulate treatment-related toxicities.

Several limitations should be acknowledged. First, this was a single-center study with a relatively limited cohort size, and the response- and toxicity-associated features identified here require validation in larger, independent cohorts. Second, the functional validation experiment was performed in the MC38 syngeneic tumor model, which does not fully recapitulate pMMR/MSS rectal cancer. Nevertheless, MC38 is an established immunocompetent colorectal tumor model for studying radiotherapy combined with immune checkpoint blockade (64, 65). In this study, MC38 was used as a proof-of-concept model to test whether GABA modulates the antitumor effect of radiotherapy plus PD-1 blockade. More molecularly relevant MSS-like models, including genetically engineered or organoid-based colorectal cancer models are needed to further validate the translational relevance of this finding (66). Third, although patients with recent antibiotic, probiotic, steroid, or immunosuppressive exposure were excluded, and covariate-adjusted models were performed where possible, residual confounding from supportive care medications, diet, lifestyle and other clinical factors cannot be excluded. Fourth, metabolite annotations from untargeted metabolomics require confirmation by targeted quantitative assays. Fifth, the random forest, receiver operating characteristic, and multiple linear regression analyses were exploratory and should be interpreted as feature-prioritization or risk-stratification approaches rather than clinically deployable prediction models. Finally, although our data nominate *K. pneumoniae* and K00128-related microbial functional signals in association with GABA, bacterial colonization, strain-level GABA production assays, or isotope-tracing experiments were not performed. Therefore, the microbial source and proportional contribution of GABA remain to unsolved. Future studies integrating validated metabolite quantification, strain-level microbial experiments, and independent clinical cohorts will be needed to translate these findings into precision iTNT management.

## Data Availability

The datasets presented in this study can be found in online repositories. The names of the repository/repositories and accession number(s) can be found in the article/**Supplementary Material**.
