# Supplementary material for: Fecal microbiome and metabolome dynamics during immunotherapy-based total neoadjuvant therapy in rectal cancer: associations with treatment response and toxicity
Source: Front Immunol. 2026 Jun 24;17:1871586. doi: 10.3389/fimmu.2026.1871586 (PMC13341674; doi:10.3389/fimmu.2026.1871586)
Supplement: Supplementary file 1 [file DataSheet1.pdf]

Supplementary Material

1 Supplementary Data

Supplementary Figure 1. Gut microbiome comparison between treatment-sequence arms across sampling timepoints
Supplementary Figure 2. Longitudinal species-level microbial changes during iTNT.
Supplementary Figure 3. Associations between longitudinally altered microbial taxa and metabolites.
Supplementary Figure 4. Response-associated species, microbial–metabolic correlations, and exploratory ROC analyses.
Supplementary Figure 5. Flow cytometry gating strategies.
Supplementary Figure 6. Representative flow cytometry plots for systematic T-cell phenotypes.
Supplementary Figure 7. Microbial diversity analyses according to treatment-related toxicity severity.
Supplementary Figure 8. Metabolite features selected in covariate-adjusted models for hematologic toxicity indices.

Supplementary Table 1. Details of antibodies used for flow cytometry.
Supplementary Table 2. Sample distribution and gut microbiome comparison between TORCH treatment-sequence arms across sampling timepoints.
Supplementary Table 3. Metabolites that significantly changed over the course of iTNT.
Supplementary Table 4. Partial Mantel analyses for toxicity-associated microbial features.
Supplementary Table 5. Metabolites with significant correlations of hematological toxicities.
Supplementary Table 6. Original and covariate-adjusted coefficients of metabolite features in hematologic toxicity-related multivariable linear regression models.
Supplementary Table 7. Comparison of gut microbiome and metabolome studies in LARC neoadjuvant treatment cohorts.
Supplementary Table 8. Fecal metabolomic profiling datasets. (Sheet 1 contains the baseline metabolite intensity matrix; Sheet 2 contains the batch-effect-corrected metabolite intensity matrix across three longitudinal timepoints).

2 Supplementary Figures

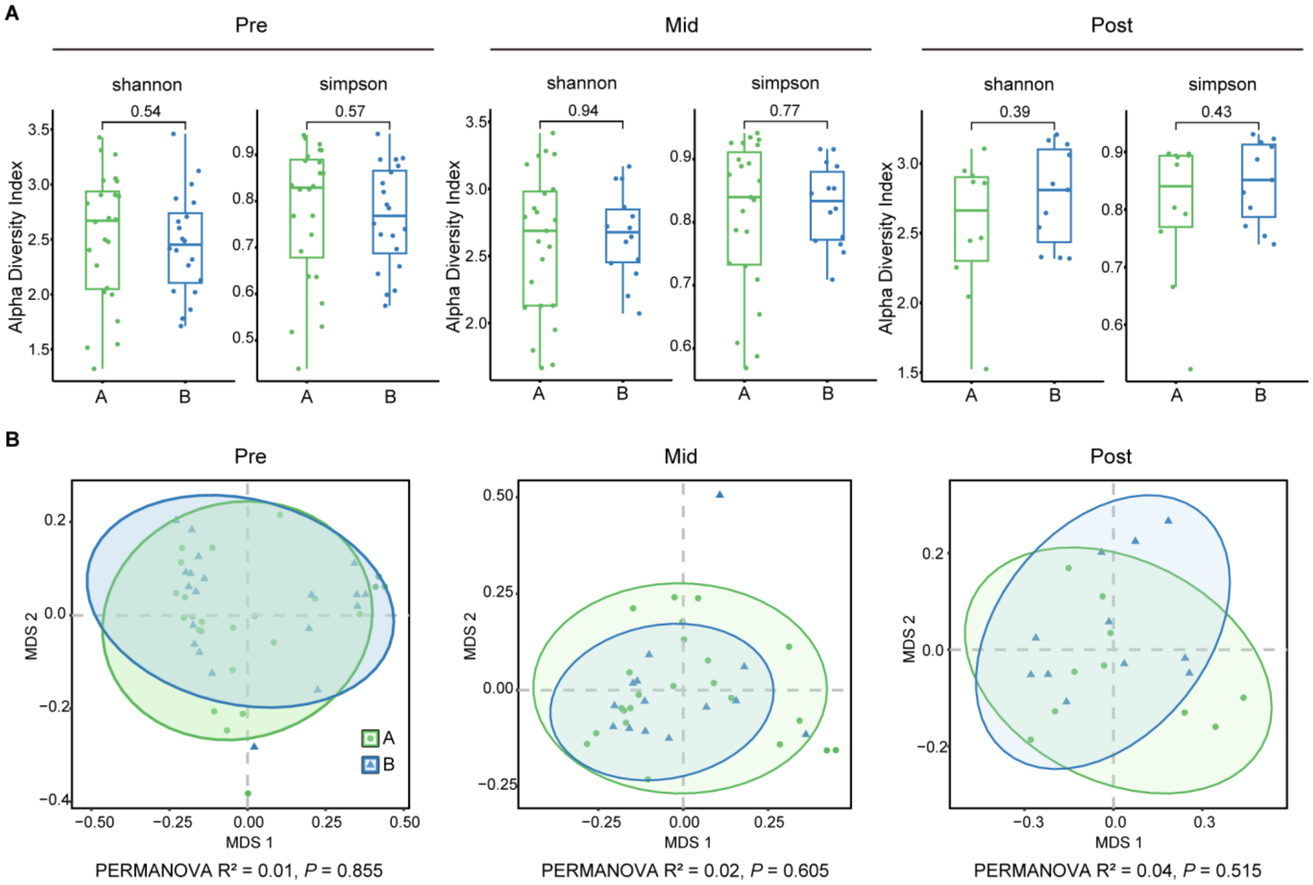

Supplementary Figure 1. Gut microbiome comparison between treatment-sequence arms across sampling timepoints. **(A)** Alpha diversity, including Shannon and Gini–Simpson indices, was compared between Arm-A and Arm-B at the pre-treatment, mid-treatment, and post-treatment timepoints. Statistical comparisons were performed using Wilcoxon rank-sum tests. **(B)** Bray-Curtis dissimilarity-based NMDS plots showing gut microbiome community structure between Arm-A and Arm-B at each timepoint. Group differences were assessed using PERMANOVA. Arm-A: Consolidation sequence; Arm-B: Induction sequence.

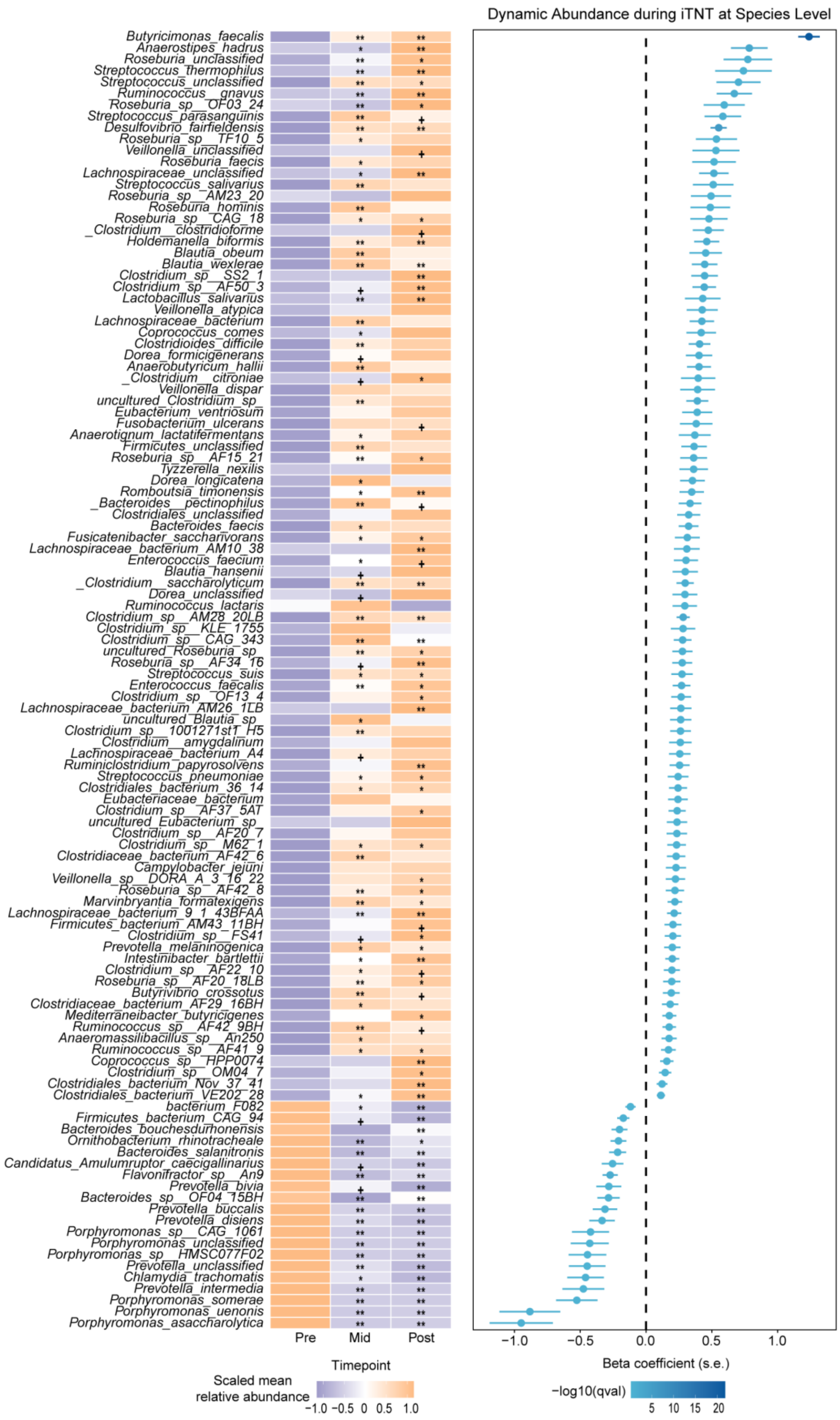

Supplementary Figure 2. Longitudinal species-level microbial changes during iTNT.

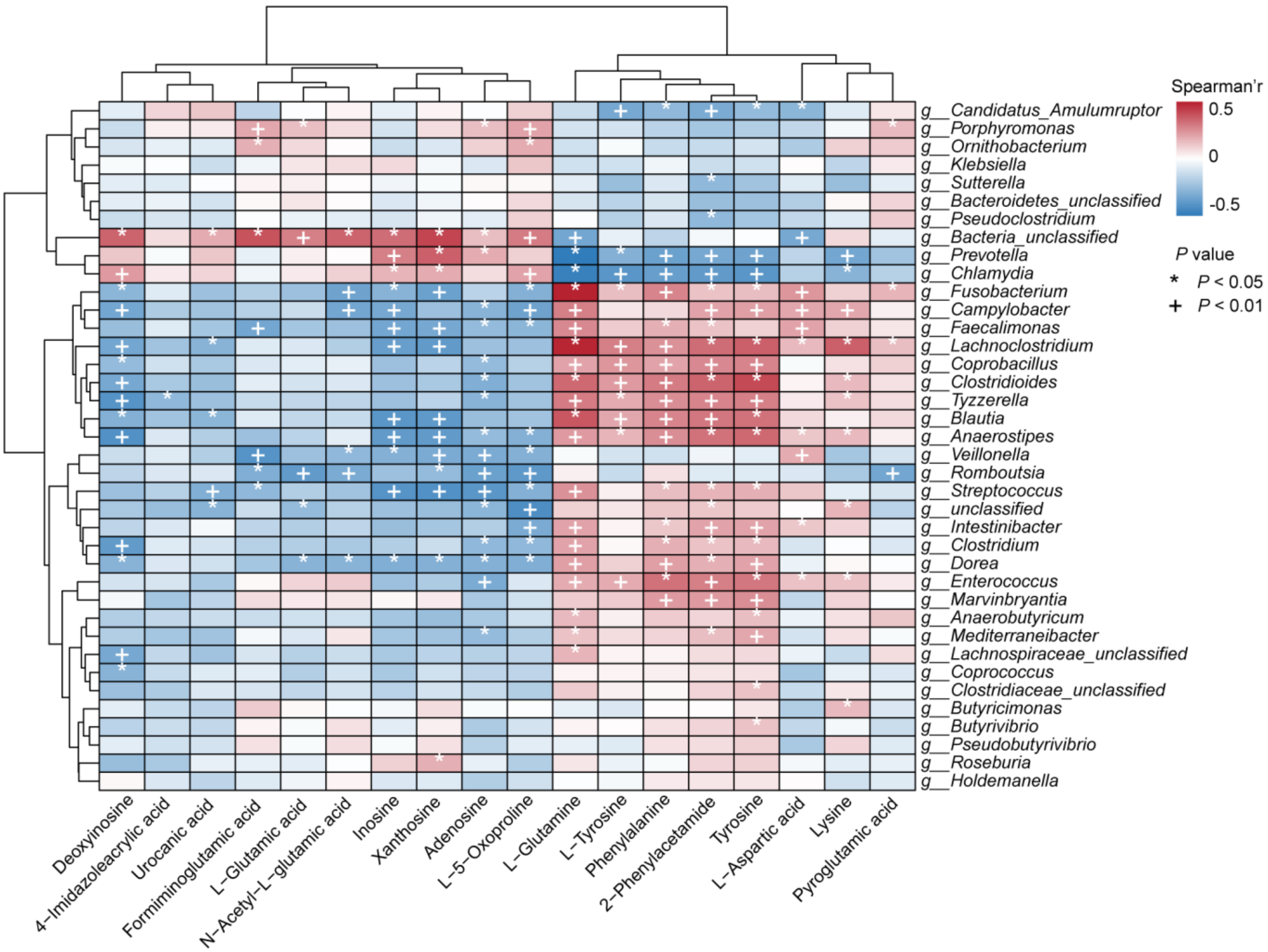

Supplementary Figure 3. Associations between longitudinally altered microbial taxa and metabolites.

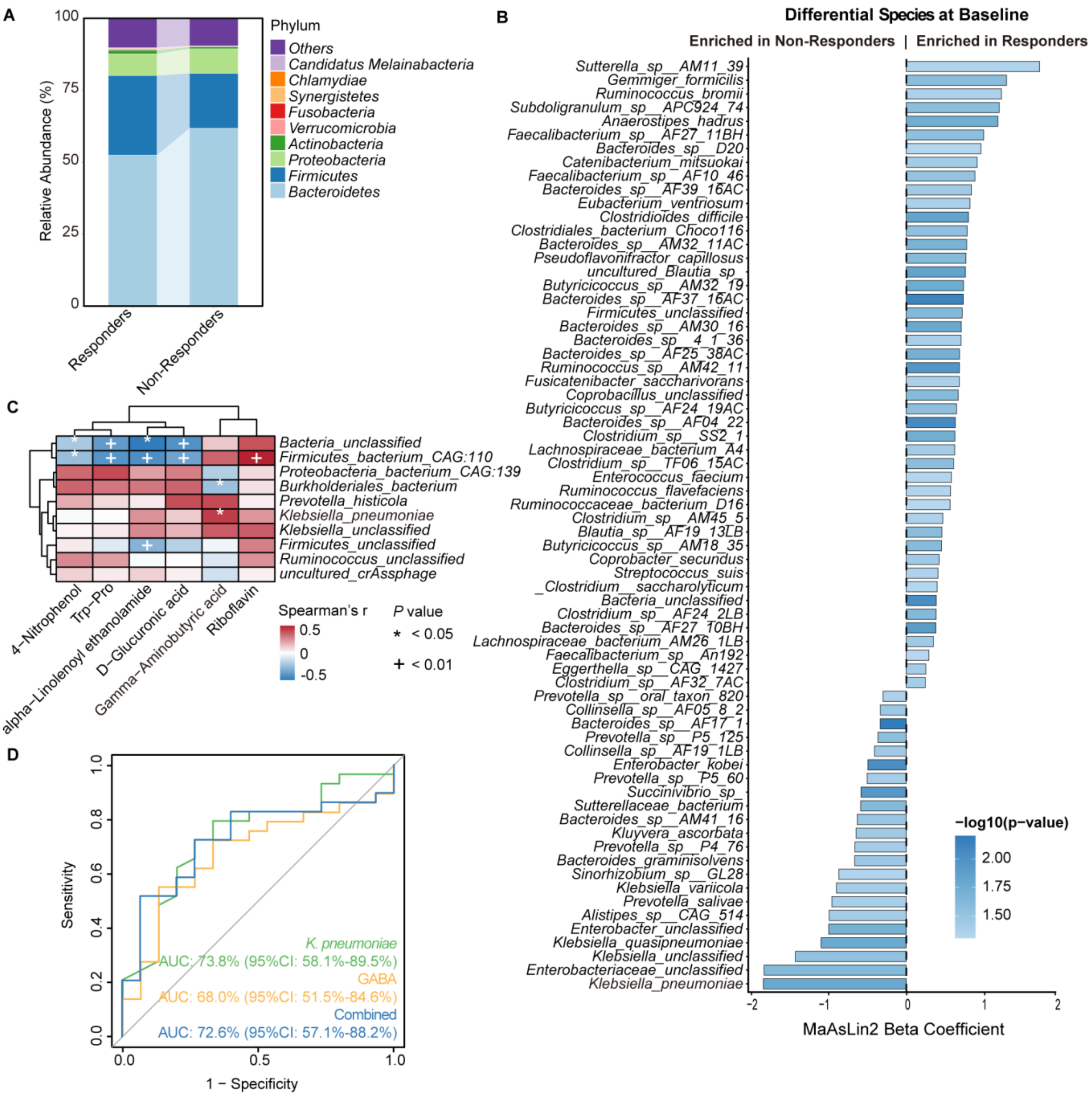

Supplementary Figure 4. Response-associated species, microbial–metabolic correlations, and exploratory ROC analyses. **(A)** Phylum-level composition of baseline fecal samples from responders and non-responders. **(B)** Differentially abundant species between responders and non-responders. **(C)** Correlation heatmap between RF-selected microbial features and RF-selected metabolic features. **(D)** Exploratory ROC curves for baseline *K. pneumoniae* abundance, GABA level, and their combined logistic regression score in relation to therapeutic response status. AUC values are shown.

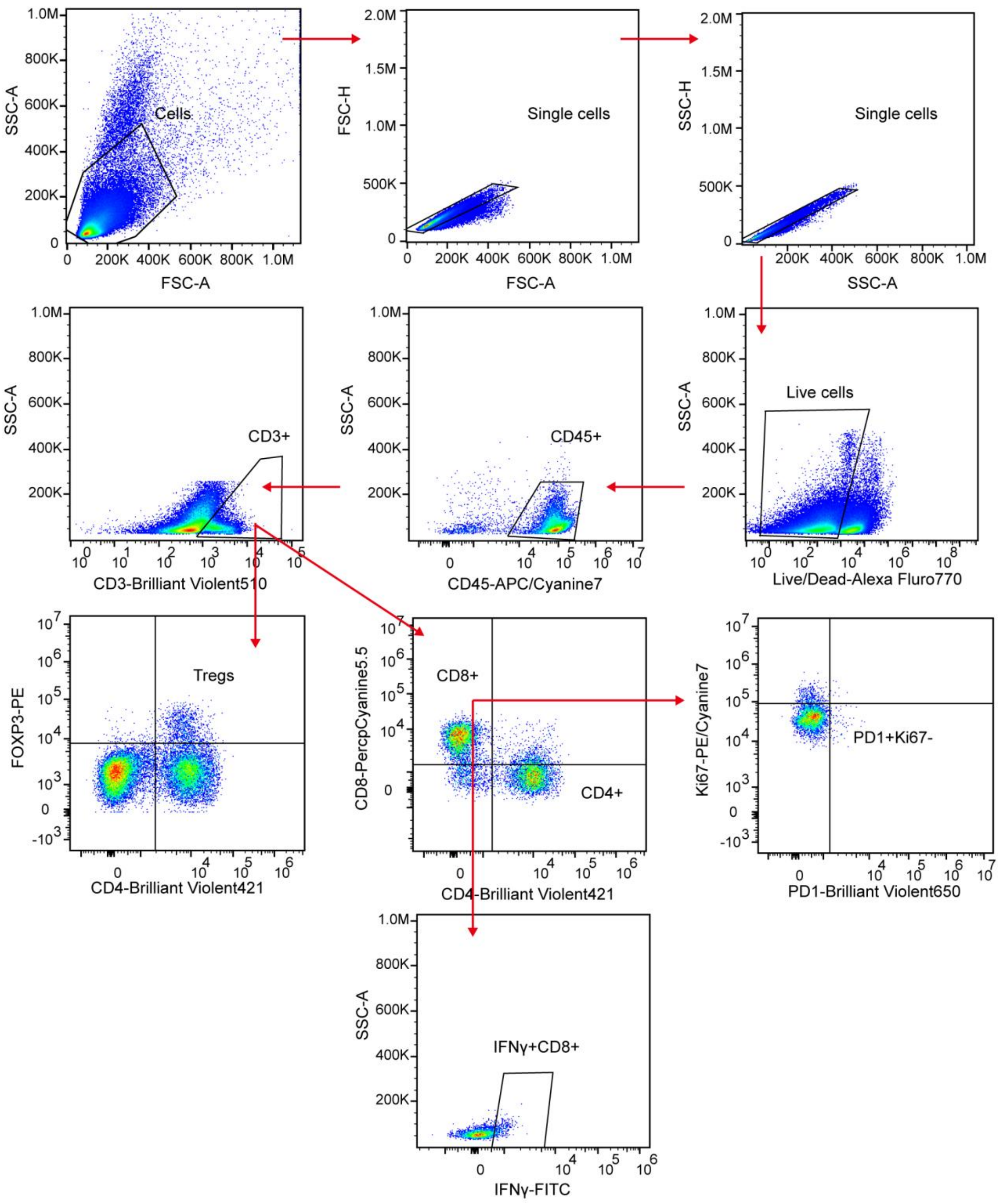

Supplementary Figure 5. Flow cytometry gating strategies.

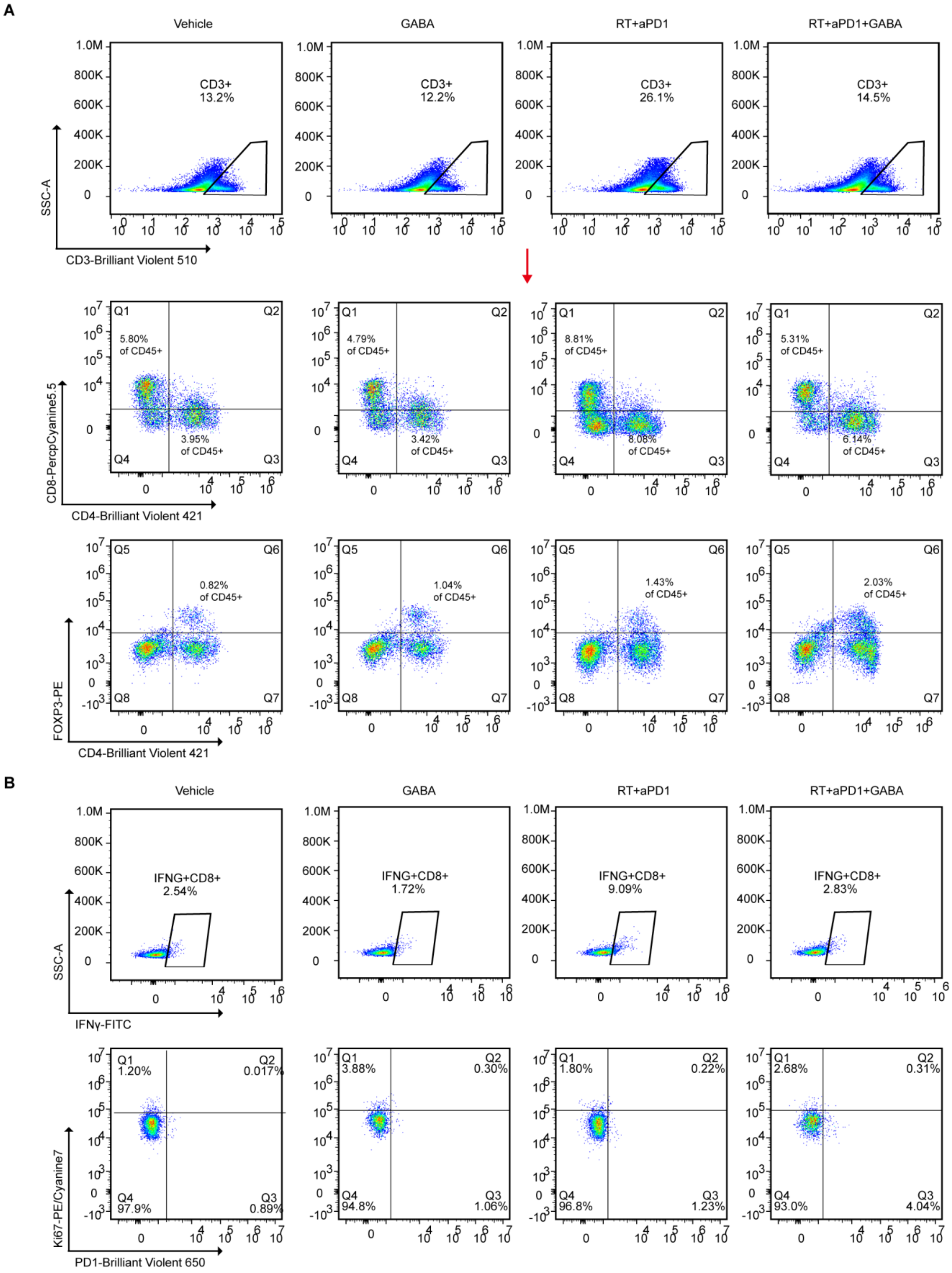
A
Vehicle
GABA
RT+aPD1
RT+aPD1+GABA
CD3+ 13.2%
CD3+ 12.2%
CD3+ 26.1%
CD3+ 14.5%
SSC-A
CD3-Brilliant Violent 510
5.80% of CD45+
3.95% of CD45+
4.79% of CD45+
3.42% of CD45+
8.81% of CD45+
8.08% of CD45+
5.31% of CD45+
6.14% of CD45+
CD8-PercpCyanine5.5
CD4-Brilliant Violent 421
0.82% of CD45+
1.04% of CD45+
1.43% of CD45+
2.03% of CD45+
FOXP3-PE
CD4-Brilliant Violent 421
B
Vehicle
GABA
RT+aPD1
RT+aPD1+GABA
IFNG+CD8+ 2.54%
IFNG+CD8+ 1.72%
IFNG+CD8+ 9.09%
IFNG+CD8+ 2.83%
SSC-A
IFNγ-FITC
Q1 1.20%
Q2 0.017%
Q4 97.9%
Q3 0.89%
Q1 3.88%
Q2 0.30%
Q4 94.8%
Q3 1.06%
Q1 1.80%
Q2 0.22%
Q4 96.8%
Q3 1.23%
Q1 2.68%
Q2 0.31%
Q4 93.0%
Q3 4.04%
Ki67-PE/Cyanine7
PD1-Brilliant Violent 650

Supplementary Figure 6. Representative flow cytometry plots for systematic T-cell phenotypes. **(A)** Representative plots showing CD3^{+} T cells, CD4^{+} T cells, CD8^{+} T cells, and Tregs. CD4/CD8 plots were displayed after gating on CD3^{+} T cells, while the indicated percentages for CD4^{+}, CD8^{+}, and Treg cells were calculated as frequencies among CD45^{+} leukocytes using the full gating hierarchy. **(B)** Representative plots showing IFNγ-producing CD8^{+} T cells and PD-1^{+}Ki-67^{-} exhausted CD8^{+} T cells. Quantitative results are shown in Figure 4E and 4F.

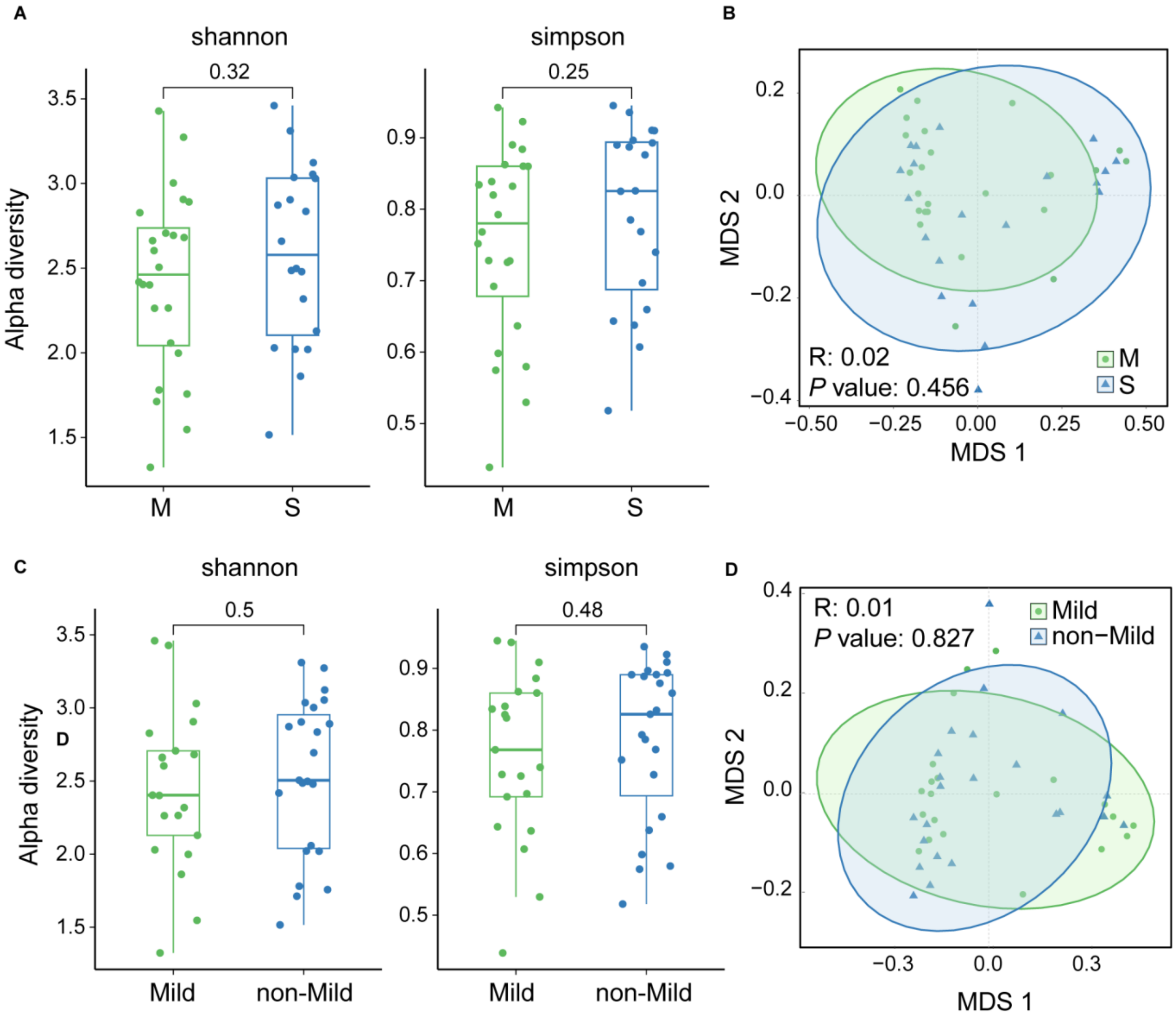

Supplementary Figure 7. Microbial diversity analyses according to treatment-related toxicity severity. **(A)** Alpha diversity, assessed by Shannon and Simpson indices at the genus level, did not differ between hematologic toxicity severity groups. M, mild-to-moderate hematologic toxicity; S, severe hematologic toxicity. **(B)** NMDS based on Bray–Curtis distances at the genus level showed no clear separation between hematologic toxicity severity groups (PERMANOVA, P = 0.456). **(C)** Alpha diversity, assessed by Shannon and Simpson indices at the genus level, did not differ between diarrhea severity groups. **(D)** NMDS based on Bray–Curtis distances at the genus level showed no clear separation between diarrhea severity groups (PERMANOVA, P = 0.827).

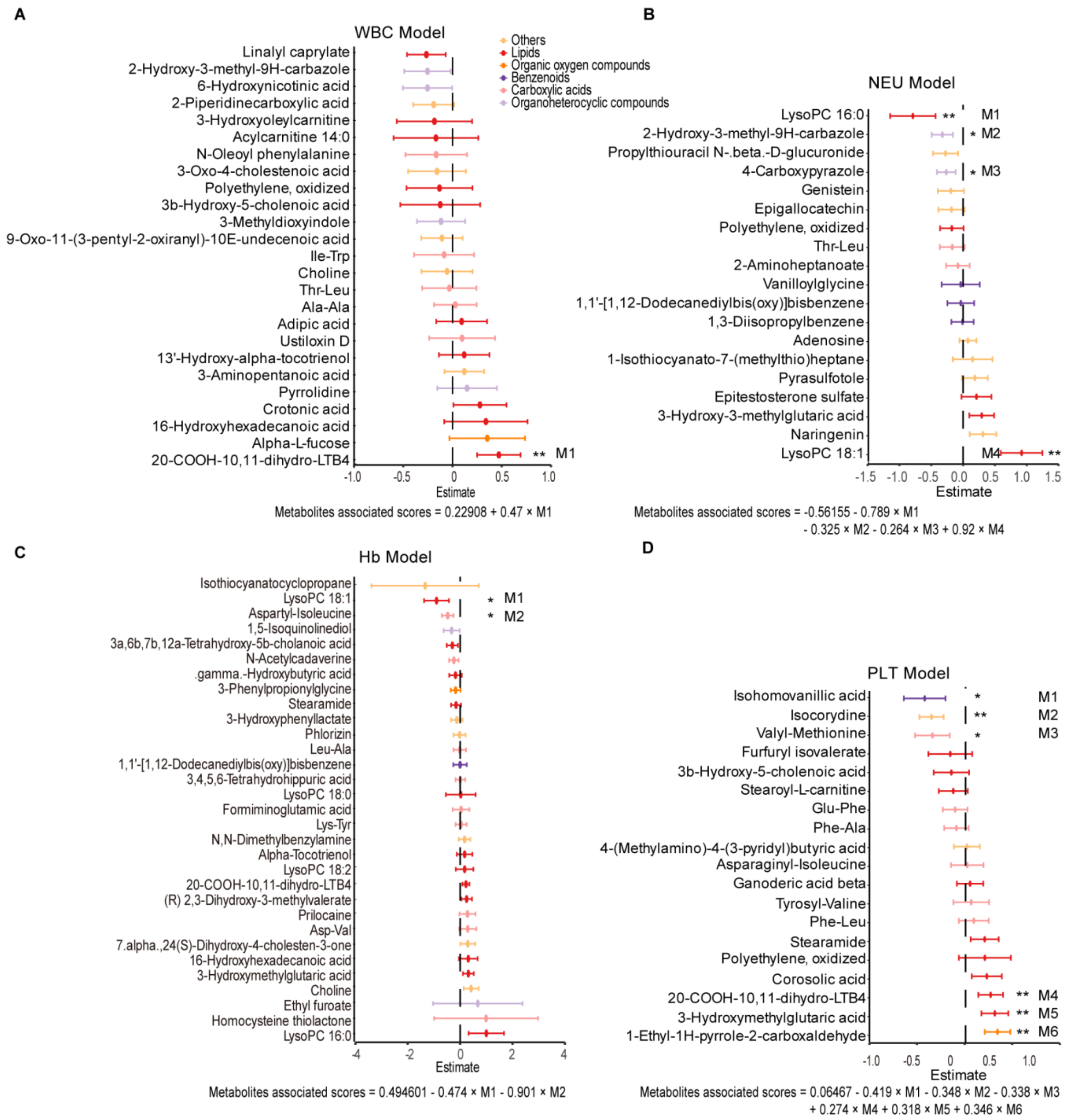

Supplementary Figure 8. Metabolite features selected in covariate-adjusted models for hematologic toxicity indices. **(A)** Metabolite features associated with the lowest white blood cell count. **(B)** Metabolite features associated with the lowest neutrophil count. **(C)** Metabolite features associated with the lowest hemoglobin level. **(D)** Metabolite features associated with the lowest platelet count. Forest plots show adjusted β coefficients and 95% confidence intervals of selected metabolite features from multiple linear regression models. Models were adjusted for the corresponding baseline blood count, treatment cycle completion, and documented treatment modification. Original and adjusted β coefficients and P values are provided in Supplementary Table 6.
